# Metabolites Secreted by Bovine Embryos In Vitro Predict Pregnancies That the Recipient Plasma Metabolome Cannot, and Vice Versa

**DOI:** 10.3390/metabo11030162

**Published:** 2021-03-11

**Authors:** Enrique Gomez, Nuria Canela, Pol Herrero, Adrià Cereto, Isabel Gimeno, Susana Carrocera, David Martin-Gonzalez, Antonio Murillo, Marta Muñoz

**Affiliations:** 1 Centro de Biotecnología Animal, Servicio Regional de Investigación y Desarrollo Agroalimentario, 33394 Gijon, Spain; imgimeno@serida.org (I.G.); scarrocera@serida.org (S.C.); davydmg@hotmail.com (D.M.-G.); antoniomurillovet@gmail.com (A.M.); mmunoz@serida.org (M.M.); 2 Centre for Omic Sciences, Eurecat, Centre Tecnològic de Catalunya (Joint Unit Eurecat- Universitat Rovira i Virgili), Unique Scientific and Technical Infrastructure (ICTS), 43204 Reus, Spain; nuria.canela@eurecat.org (N.C.); pol.herrero@eurecat.org (P.H.); adria.cereto@ce.eurecat.org (A.C.); 3 Carrera de Medicina Veterinaria, Facultad de Ciencias Pecuarias, Escuela Superior Politécnica de Chimborazo, Riobamba EC060150, Ecuador

**Keywords:** bovine, embryo, in vitro, recipient, pregnancy, metabolism

## Abstract

This work describes the use of mass spectrometry-based metabolomics as a non-invasive approach to accurately predict birth prior to embryo transfer (ET) starting from embryo culture media and plasma recipient. Metabolomics was used here as a predictive platform. Day-6 in vitro produced embryos developed singly in modified synthetic oviduct fluid culture medium (CM) drops for 24 h were vitrified as Day-7 blastocysts and transferred to recipients. Day-0 and Day-7 recipient plasma (*N* = 36 × 2) and CM (*N* = 36) were analyzed by gas chromatography coupled to the quadrupole time of flight mass spectrometry (GC-qTOF). Metabolites quantified in CM and plasma were analyzed as a function to predict pregnancy at Day-40, Day-62, and birth (univariate and multivariate statistics). Subsequently, a Boolean matrix (F1 score) was constructed with metabolite pairs (one from the embryo, and one from the recipient) to combine the predictive power of embryos and recipients. Validation was performed in independent cohorts of ETs analyzed. Embryos that did not reach birth released more stearic acid, capric acid, palmitic acid, and glyceryl monostearate in CM (i.e., (*p* < 0.05, FDR < 0.05, Receiver Operator Characteristic—area under curve (ROC-AUC) > 0.669)). Within Holstein recipients, hydrocinnamic acid, alanine, and lysine predicted birth (ROC-AUC > 0.778). Asturiana de los Valles recipients that reached birth showed lower concentrations of 6-methyl-5-hepten-2-one, stearic acid, palmitic acid, and hippuric acid (ROC-AUC > 0.832). Embryonal capric acid and glyceryl-monostearate formed F1 scores generally >0.900, with metabolites found both to differ (e.g., hippuric acid, hydrocinnamic acid) or not (e.g., heptadecanoic acid, citric acid) with pregnancy in plasmas, as hypothesized. Efficient lipid metabolism in the embryo and the recipient can allow pregnancy to proceed. Changes in phenolics from plasma suggest that microbiota and liver metabolism influence the pregnancy establishment in cattle.

## 1. Introduction

Developing “a robust, non-invasive test with which to select single embryos for transfer into the uterus” remains one of the major challenges for in vitro embryo technologies [1] to predict embryo quality and pregnancy outcome [2]. The interest of identifying viable embryos increases as embryonic competence decreases, since embryo transfer (ET) with the less competent embryos means using more recipients per born calf. Furthermore, since embryo cryopreservation reduces to-term embryonic viability by triggering pregnancy losses at different gestational endpoints [3,4,5,6,7,8]. The interest in predicting pregnancy with cryopreserved embryos is higher than within fresh ones.

In bovine reproduction, predictive studies typically selected either embryos or recipients for pregnancy and birth, while the other factor of the pregnancy equation was assumed to be viable (the embryo) or competent (the recipient). The need for extracting information from both embryo and recipient to accurately profile pregnancy was stated almost two decades ago [8], but no solution has yet leveraged the predictive power of both sources in a single tool.

The evaluation of embryonic quality for ET requires individual assessment and minimal damage to the embryo. Thus, collection of embryonic cells by biopsy allows the investigation of chromosomal stability [9] and the expression of genes regulated in single embryos with higher competence to develop [10,11]. Biopsy is, nevertheless, invasive, and the damage done to the embryo may reduce pregnancy rates, potentially leading to misidentification of embryos with actual pregnancy potential [10,12].

In contrast to biopsy, non-invasive techniques may select competent embryos with morphology and the development stage as the most used in practice. However, these criteria are subjective, and the resultant evaluation judgements within in vitro produced (IVP) embryos are far from unanimous [8,13]. Oxygen consumption [14,15], which can be coupled with time-lapse monitoring of embryo morphology and development kinetics, was efficient in predicting pregnancy, even though sophisticated equipment is required [16,17]. The above techniques are complex, expensive, and time-consuming, and require skilled operators. Therefore, simpler and non-invasive techniques are necessary to identify developmentally competent embryos.

Within non-invasive procedures, analysis of single culture medium (CM) can identify molecules released and consumed by the embryo. Metabolites, proteins, and the cargo of extracellular vesicles (EV), as small RNAs released by the embryo, are detectable in the spent CM and are informative of embryonic traits [18,19,20,21]. However, the association of such molecules with pregnancy has been investigated mainly in human embryos [19,22,23] instead of in cattle.

Metabolomics encompasses a group of sensitive techniques for broad analysis of the whole metabolome in any species. Metabolomics can gather information from genomes and the environment for improved definition of a given phenotype. One of the most interesting applications of metabolomics is the ability to predict (as opposed to diagnose) a physiological or pathological state [24,25]. Within cattle embryos, metabolomic studies were mainly focused on predicting embryonic sex [26,27,28,29] or short-term development landmarks [30,31,32], while few studies predicted pregnancy endpoints [6,33]. 

On the recipient side, the usual selection procedures based on estrus detection, corpus luteum exploration, and/or progesterone concentration measurement exclude animals that could reach pregnancy [34,35,36]. Metabolomics can define the temporal receptivity for pregnancy of a particular recipient [6,33,37,38]. However, we were unable to combine the predictive potential of embryos and recipients by using Fourier transform infrared spectroscopy analysis of CM and plasma [6,33]. Other authors analyzed the endometrial gene expression of the recipient in the cycle that precedes ET using invasive procedures [10,39].

The optimal predictable gestational endpoint is birth. However, recipient biomarkers predict pregnancy more effectively at Day-62 and not at birth, in particular with vitrified/warmed (V/W) embryos [37,38]. Metabolomics, and eventually proteomics, as technologies closer to the phenotype than genomics [25,40], can better define the temporal receptivity of a particular recipient within a cycle. Developing systematic and reliable methods for recipient and embryo selection is, therefore, a challenging and novel objective for cattle ET technology, particularly if embryos are cryopreserved by vitrification. We hypothesized that the pregnancy predictions normally obtained with separate metabolomics analyses could be increased with the appropriate combination of biomarkers from embryo CM and recipient plasma. In this study, using gas chromatography coupled to the quadrupole time of flight mass spectrometry (GC-qTOF), we, for the first time, identified biomarkers in recipient plasma and embryo CM that efficiently predicted pregnancy viability by combining information from the embryo and the recipient sides.

## 2. Results

In the discovery study, all conditioned medium (CM) and plasma samples were analyzed, but one Day-0 plasma sample was not collected. The gas chromatography coupled to quadrupole time of flight mass spectrometry (GC-qTOF) analysis led to identification and quantification of *N* = 37 metabolites in CM (Supplemental Table S1) and *N* = 71 metabolites in plasma (Supplemental Table S2). A complete description of each ET performed in this study and its gestational trajectory is shown in Supplemental Table S3.

### 2.1. Breed Effects on Embryo Culture Medium and Recipient Plasma

Within discriminant analysis, the metabolomes from embryo CM (Supplementary Figure S1A,B) and Day-7 plasma (Supplemental Figure S1C,D) did not significantly differ between breeds. However, Day-0 plasma reflected close to significant differences between breeds by permutation with Partial Least Square-Discriminant Analysis (PLS-DA) on Day-0 (*p* < 0.10) (Supplemental Figure S1E) and appreciable separation by sparse PLS-DA (sPLS-DA) (Supplemental Figure S1F). These differences are consistent with our previous study that identified 55 regulated metabolic pathways between Asturiana de los Valles (AV) (*N* = 80 samples) and Holstein (*N* = 107 samples) [41].

### 2.2. Single Candidate Biomarker Discovery

Following the identified breed effects, candidate metabolites were analyzed as an aggregate in CM (in all samples) and separated by breed in recipient plasma from AV (*N* = 13 ETs) and Holstein (*N* = 17 ETs). Crossbred recipient samples (*N* = 6) were not considered for the breed study in plasma.

#### 2.2.1. Culture Medium

In CM, on Day-40, no metabolite fulfilled the required statistical conditions for candidate metabolites as measured by pregnancy status, but stearic acid was identified as differentially regulated in pregnancies diagnosed at Day-62 (Table 1). 

**Figure 1:**
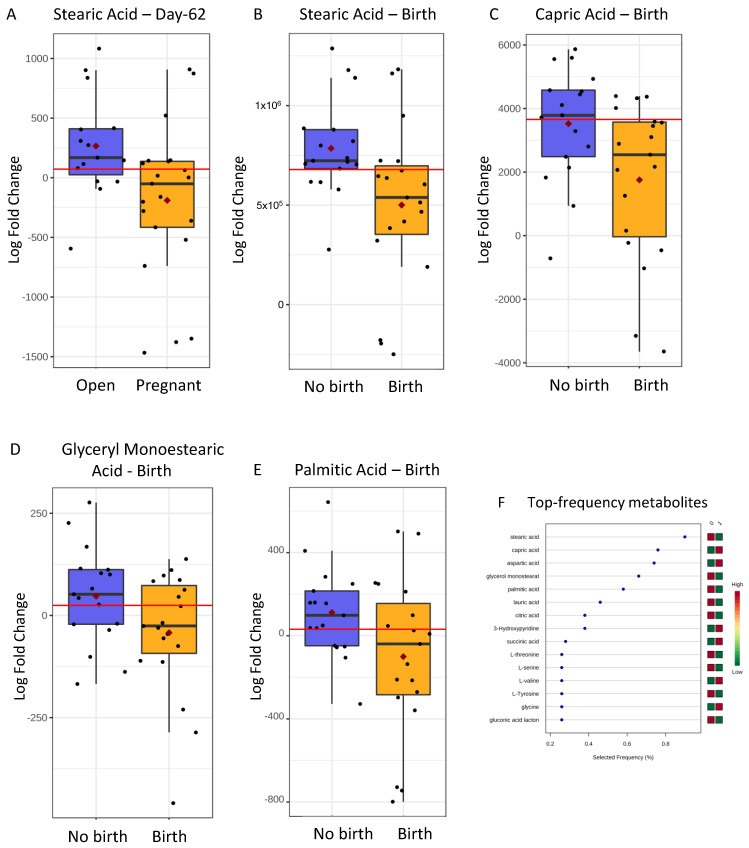
Boxplots of metabolite biomarkers of embryonic viability measured in embryo culture medium of embryos that led to pregnancy or non-pregnancy: Stearic Acid on pregnancy Day-62 (**A**; ROC-AUC = 0.733 [0.570–0.898]), and Stearic acid at birth (**B**; ROC-AUC = 0.743 [0.437–0.838]), Capric Acid at birth (**C**; ROC-AUC = 0.728 [0.465–0.840]), Glyceryl Monostearate at birth (**D**; ROC-AUC = 0.670 [0.570–0.898]), and Palmitic Acid at birth (**E**; ROC-AUC = 0.669 [0.533–0.870]). Black dots represent the concentrations of all samples of each metabolite scaled as Log Fold Change values. The notch indicates the 95% confidence interval around the median. The mean concentration of each group is shown by a red diamond. The optimal cut-off is indicated as a horizontal red line (i.e., the closest to the left-hand corner in the corresponding ROC-AUC). (**F**) represents the above and other top metabolites (Y axis) ranked by their contribution to classification accuracy in predicting birth expressed as frequency (X axis) (Random Forests multivariate classification algorithm).

Boxplots for all the metabolites that showed ROC-AUC > 0.650 and significant (*p* < 0.05) or close to significant (*p* < 0.07) value are shown in Figure 1A–E. These metabolites were also selected in the top frequency (%) ranking by Random Forests (RF) (Figure 1F) as candidate biomarkers.

#### 2.2.2. Plasma from Holstein Recipients

Metabolites that differed between plasmas from open and pregnant Holstein recipients on Day-0 were hydrocinnamic acid (Birth and Day-40), and 2-hydroxybiphenyl and glycerol-phosphate (Day-40) (Table 2). 

**Figure 2:**
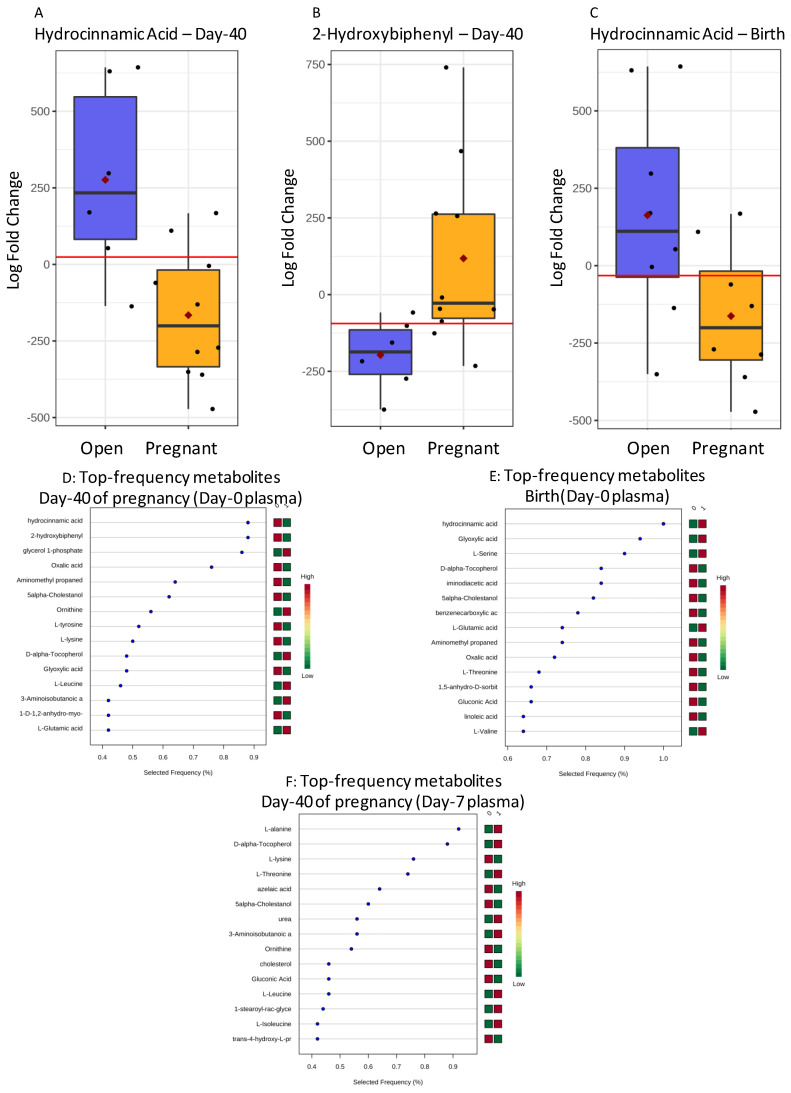
Boxplots of metabolite biomarkers of pregnancy viability measured in plasma of pregnant and open Holstein recipients on Day-0 (Hydrocinnamic Acid (**A**) and 2-Hydroxybiphenyl (**B**) at pregnancy Day-40 and Hydrocinnamic Acid at birth (**C**)). Black dots represent the concentrations of all samples of each metabolite scaled as Log Fold Change values. The notch indicates the 95% confidence interval around the median. The mean concentration of each group is shown by a red diamond. The optimal cut-off is indicated as a horizontal red line (i.e., the closest to the left-hand corner in the corresponding ROC-AUC). Frequency rankings by a Support Vector Machine (SVM) algorithm for top biomarkers, including those of the above boxplots, are shown at gestational Day-40 on Day-0 plasma (**D**) and birth (**E**), and on Day-7 at birth (**F**) (1: Pregnant or birth to term recipients. 0: open recipients).

In Day-7 plasma, alanine and lysine differed within pregnancies diagnosed at birth, while threonine differed on Day-62. Such metabolites were confirmed as candidate biomarkers with ROC-AUC > 0.778 and either significant (*p* < 0.05) or close to significant (*p* < 0.07) values. Boxplots of the most relevant plasma Holstein biomarkers from Day-0 plasma are shown in Figure 2A–C and frequency rankings by the Support Vector Machine (SVM) for Day-0 candidate biomarkers at gestational Day-40 and birth, and on Day-7 at birth, are shown in Figure 2D–F.

#### 2.2.3. Plasma from AV Recipients

In Day-0 plasma, 6-methyl-5-hepten-2-one, stearic acid, and palmitic acid (at birth). Palmitic acid (at Day-62 and Day-40, same results) differed (*p* < 0.05) or tended to differ (0.05 < *p* < 0.072) (Table 3) between diagnosed open and pregnant AV recipients. In Day-7 plasma, hippuric acid differed in pregnancies diagnosed at birth, and hippuric acid, valine, N-(2-hydroxyethyl) iminodiacetic acid 2 (abbreviated as HEIDA), and dehydroascorbic acid differed in pregnancy diagnoses at Day-62 and Day-40. These metabolites were candidate biomarkers with ROC-AUC > 0.832 and significant (*p* < 0.05) or close to significant (*p* < 0.072) values.

**Figure 3:**
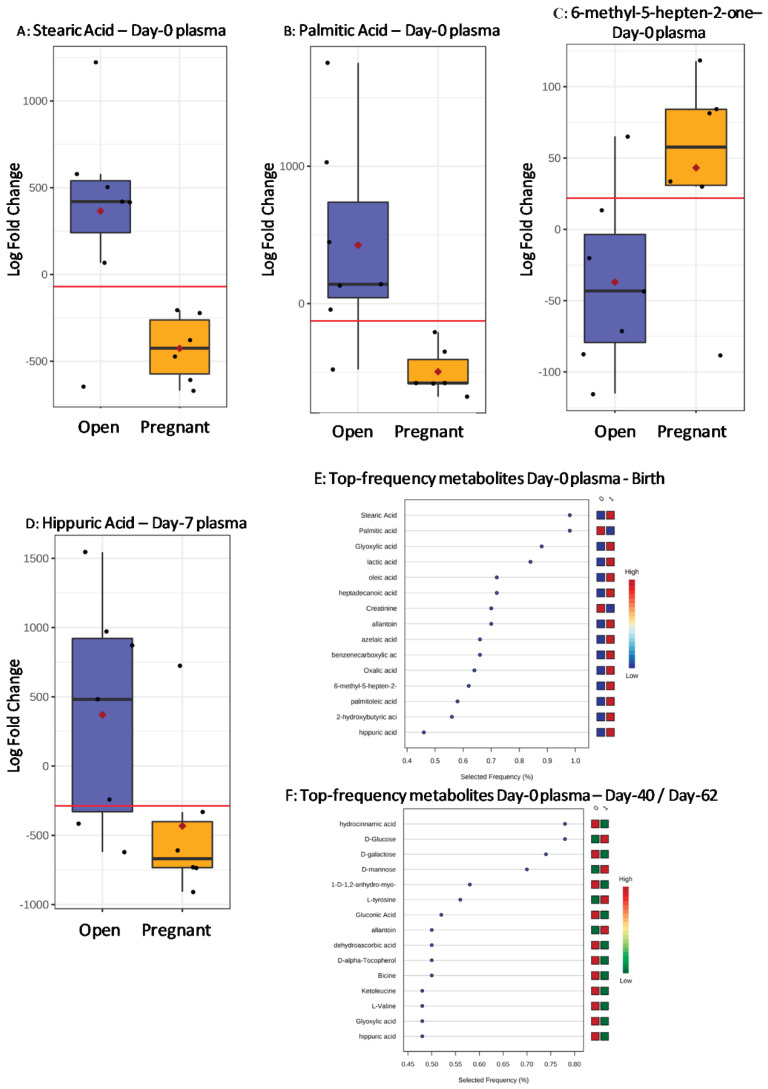
Boxplots of the biomarker metabolites measured in Day-0 plasma of Asturiana de los Valles recipients transferred with vitrified/warmed embryos (discovery study) as diagnosed at birth (i.e., Stearic Acid, ROC-AUC = 0.881 (0.571–1.000) (**A**), Palmitic Acid, ROC-AUC = 0.976 (0.786–1.000) (**B**), and 6-methyl-5-hepten-2-one, ROC-AUC = 0.833 (0.500–1.000) (**C**)), and in Day-7 plasma (Hippuric Acid, ROC-AUC = 0.857 (0.571–1.000) (**D**)), from open (blue boxes) and pregnant (yellow boxes). Black dots represent the concentrations of all samples of each metabolite scaled as Log Fold Change values. The notch indicates the 95% confidence interval around the median. The mean concentration of each group is shown by a red diamond. The optimal cut-off is indicated as a horizontal red line (i.e., the closest to the left-hand corner in the corresponding ROC-AUC). Frequency rankings by a Support Vector Machine (SVM) algorithm for top biomarkers, including those of the above boxplots, are shown on Day-0 plasma at birth (**E**), and on gestational days 40 and 62 (same biomarkers) (**F**) (1: Pregnant or birth to term recipients. 0: open recipients).

Boxplots in Figure 3 depict the most relevant metabolite biomarkers found in AV plasma at birth on Day-0 (Figure 3A–C) and on Day-7 (Figure 3D). Within the SVM ranking of selected frequencies in Day-0 plasma at birth, stearic acid, and palmitic acid, and 6-methyl-5-hepten-2-one (Figure 3E) appeared in the top place. On Day-40 and Day-62, hippuric acid, valine, bicine, and dehydroascorbic acid were among the top 15 selected metabolites (Figure 3F).

### 2.3. Combined Biomarker Discovery

Combinations of the embryo CM metabolites glyceryl monostearate, capric acid, palmitic acid, and stearic acid were examined by multivariate analysis using SVM, PLS-DA, and RF (Supplementary Figure S2) at birth. The algorithm providing higher predictive accuracy (PA) was PLS-DA when used in combinations of two metabolites. ROC curves deployed in Supplemental Figure S3 showed the best value combinations of pairs of metabolites. Such ROC values obtained for combined features were generally similar to or slightly lower than single ROC-AUC values for each CM metabolite shown in Table 1. Within AV recipients, the mean PA multivariate models included stearic acid, 6-methyl-5-hepten-2-one, and palmitic acid on Day-0 at birth. The best mean PA model was PLS-DA and included two metabolites, whose combinations resulted in ROC-AUC values between 0.857 and 0.897, similar to or slightly lower than the single ROC-AUC values shown in Table 3. Models in Holstein recipients included hydrocinnamic acid, 2-hydroxybiphenyl, and glycerol-phosphate on Day-40, which is the most representative pregnancy endpoint in Table 2. In this case, the best mean predictive model was RF with three metabolites (mean PA: 75%). The AUC-ROC value measured involving these three metabolites was 0.890, which is again similar to or lower than the single ROC-AUC values shown in Table 2. 

2.4 F1 Score: combinations of single metabolites from CM and plasma were identified by the F1 score as potential biomarkers for pregnancy and birth. F1 scores were calculated both within aggregate and separate embryonic stages on Day-6 (morula and early blastocyst) and within separate recipient breeds. Embryonic stages on Day-6 are well-controlled, fixed factors in individual embryo culture. F1 scores based on embryonic stages are shown only in the Holstein recipient discovery group (*N* = 17 samples), not in the AV discovery group because its lower sample numbers (*N* = 13) precluded these calculations. The most robust and highly significant (*p* < 0.02 to *p* < 0.001) F1 scores identified for pregnancy and birth are shown in Table 4.

F1 scores based on embryonic stages are shown within the Holstein discovery group (*N* = 16 and *N* = 17 sample pairs from Day-0 and Day-7, respectively), and were not possible in the AV discovery group because of lower N (*N* = 13 samples).

Probability values within F1 scores were calculated as chi-square Manzel-Haenszel correction. Two single biomarkers identified in CM (capric acid and glyceryl-monostearate) gave the best combinations with biomarkers in plasma. These plasma biomarkers forming the F1 score were mostly identified before in our single biomarker study (Section 2.2.1, Section 2.2.2 and Section 2.2.3) with few exceptions (e.g., heptadecanoic acid in AV, citric acid in Holstein). As hypothesized, distinguishing by embryonic stage on Day-6 generally led to a higher F1 score than with an aggregate Day-6 embryonic stage. At the same time, with few exceptions, the F1 score often showed higher predictive potential than the individual ROC-AUC value of each metabolite considered and/or aggregation of several metabolites from either embryonal or recipient origin. An example of how the F1 score works for combined biomarker calculation is given in Supplemental Table S4.

### 2.4. Candidate Biomarker Validation

Using a targeted biomarker approach, we analyzed the selected candidate biomarkers in an independent cohort of Holstein recipients (*N* = 19 ETs). Plasma samples collected on Day-0 were preferred for validation owing to the interest in anticipating the time to select ET recipients in the field by seven days. Metabolite biomarkers analyzed were chosen by their best values forming F1 scores (i.e., capric acid and glyceryl-monostearate in CM), and hydrocinnamic acid, citric acid, and hippuric acid in Day-0 plasma). Palmitic acid was discarded because its levels in CM were around the detection limit. Supplemental Table S5 shows relative concentrations of embryos transferred, grouped by Day-6 stage and culture medium as related to birth, and the major fixed effects identified. No significant differences were observed. However, the *P* value for F1 was significant within all metabolite combinations as measured by the Day-6 embryonic stage, as shown in Table 5.

## 3. Discussion

In this work, we identified metabolites by non-invasive methods in the embryo CM that represent predictive biomarkers of pregnancy and birth in cattle. We also found candidate metabolite biomarkers in recipient plasma whose numbers were more abundant on Day-0 than Day-7, in contrast with recent ^1^H-NMR studies that identified all biomarkers on Day-7 [37,38]. The combined use of single metabolites from the embryo and the recipient increased the predictive power, as hypothesized, indicating that the assumption of full viability of the partner embryo or the recipient confounds pregnancy estimation [8]. Such improved predictions of the F1 score were also superior to combinations of metabolites either from the embryo or from the recipient alone. Collectively, the F1 score showed a robust tool for pregnancy prediction.

### 3.1. Embryo Metabolites

Non-viable embryos released higher amounts of non-esterified saturated fatty acids (NEFAs) (stearic, capric, and palmitic acids) and glyceryl-monostearate into the CM. The lipid metabolism was, therefore, highly involved in the ability of V/W embryos to reach pregnancy to term. Lipid stocks in embryonic cells consist mainly of granules of triglyceride [42,43] that decrease from the morula to the expanded blastocyst stage [44,45]. At the same time, lipolytic gene expression increases [45]. With independence of the lipid load, lipid breakdown mechanisms are conserved and active in mammalian embryos [42,46] including cattle [47] and early embryos that are able to develop in the absence of exogenous substrates (reviewed by Reference [48]). Furthermore, bovine IVP embryos can use products of beta oxidation as energy substrates [49,50] and synthesize steroids and hormones from lipids [47]. The excess of embryonic NEFA released into CM could be a protective mechanism, since high saturated FA concentrations are detrimental for oocyte development and embryo quality [51,52,53,54], whereas unsaturated fatty acids (FAs) are protective [52,55,56,57,58]. Thus, poor quality embryos that do not reach full-term pregnancy would not be able to oxidize an excess of FAs and would release them.

Many studies have expressed the detrimental effects of lipid stored in embryonic cells to survive cryopreservation [58,59,60]. However, recent findings suggest that the lipid contents can be related with overall embryonic viability rather than strict survival to cryopreservation [61,62]. We observed similar evidence with V/W embryos cultured free of protein for 24 h, [44,59], whereby lipid stocks decrease and the transferred blastocysts did not undergo the high miscarriage rates shown by their counterparts produced with protein [59]. Our present results confirm this hypothesis, since the fully viable embryos were those bearing lower lipid degradation (i.e., less active lipid catabolism), as shown by the reduced FA contents in their spent media. To our knowledge, no other similar metabolomic study associated with the embryo and its viability to term in a non-invasive way.

### 3.2. Recipient Plasma Metabolites

Candidate biomarkers were detected at more than one pregnancy endpoint. Thus, cows that sustained birth to term showed lower concentrations of hydrocinnamic (Holstein) and hippuric (AV) acids. Such phenol-related metabolites predicted pregnancy in Day-0 plasma in Holsteins, (i.e., hydrocinnamic acid (on Day-40 and birth, and 2-hydroxybiphenyl on Day-40), and in Day-7 plasma in AV (i.e., hippuric acid, all pregnancy endpoints). In contrast, hippuric acid was found to not be pregnancy predictive in a ^1^H NMR study with Holstein and AV recipient plasma [37,38], which is controversial and requires further research. Ruminal microorganisms reduce forage phenolic monomers to phenyl-acids, such as hydrocinnamic acid, which is metabolized to hippuric acid in the liver [63]. Hydroxybiphenyl related compounds can be degraded in the rumen by bacteria through the biphenyl catabolic pathway [64,65]. However, increased 2-hydroxybiphenyl in pregnant recipients is surprising since it is a fungicide that would be more related to feeding than to the endogenous cow metabolism. The influence of phenolics–and the participation of ruminal microbiota–on pregnancy merit further research.

The regulation of plasma amino-acids within pregnancy prediction was generally very consistent with previous findings by ^1^H-NMR [37,38]. Thus, in Holsteins, l-threonine, l-alanine, l-Lysine, and 3-methyl-2-oxovalerate, which is an l-isoleucine metabolite, were pregnancy predictive with fresh or V/W embryos [37]. Furthermore, l-lysine, l-alanine, and l-threonine were in the top frequencies of SVM and RF [37,38]. In agreement with our results, a similar ^1^H NMR study within V/W embryos in the AV breed, showed L-valine to be pregnancy predictive for Day-40/Day-62, while L-isoleucine predicted term pregnancy [38]. Such changes in l-Valine and l-Isoleucine plasma levels must be interpreted with caution, as their concentrations might be altered by dietary protein [66], lactation [67], and bacterial lipopolysaccharide [68].

Metabolites involved in lipid metabolism likely represent the major differences detected in plasma between pregnant and open recipients in this work. Holstein pregnant recipients showed higher concentrations of glycerol-3-phosphate (G3P), which controls triacylglycerol synthesis together with fatty acyl CoA, substrate availability, and insulin as a promoter [69]. In addition, the genes that regulate lipogenesis are active in non-lactating cows [69,70], and the moderate increase in G3P in Holstein recipients that were later pregnant on Day-40 points out that satisfied energy requirements sustain early pregnancy.

In AV recipients, stearic acid, palmitic acid, dehydroascorbic acid (DHA), and 6-methyl-5-hepten-2-one differed in concentration in Day-0 and Day-7 plasma between pregnant and open recipients. The increase of NEFAs in blood is associated with lipid degradation in the cow as shown within cows in negative energy balance [71,72,73]. Stearic and palmitic acids alter the endometrial cell function by promoting lipid deposition and pro-inflammatory gene expression [74,75,76]. Furthermore, as shown above within embryos, an unsaturated NEFA-rich environment is negative for embryo development and viability. Concentrations of stearic acid were lower in recipients that reached birth, and palmitic acid was lower in recipients at all pregnancy endpoints analyzed. We have already observed the same trend in another study with Holstein recipients that become pregnant after receiving V/W embryos [37]. In an AV study [38], 2-hydroxybutyric acid and acetone were decreased in recipients that reached birth. These findings show the main role of lipid metabolism, consistent with less lipid breakdown metabolites in the blood of pregnant recipients.

Another interesting metabolite is DHA, which may act through two mechanisms in the recipient. First, as an enzyme cofactor for the synthesis of carnitine, neutralizing the reactive oxygen species arising from FA mitochondrial metabolism [77]. Second, directly on the embryo, by increasing E_2_ production, as shown in human cytotrophoblast cells [78], through which it may have a role in pregnancy. In the cow, vitamin C requirements can be satisfied with the ascorbic acid (AA) synthesis from glucose. AA may, in turn, be metabolized to DHA [79]. Therefore, in our work, the lower DHA levels in pregnant AV recipients are consistent with the lower availability of NEFA and lower need for DHA itself.

### 3.3. Pregnancy Prediction: Single Biomarkers

Significant fold changes within ROC-AUC values in embryos were all >2, while in recipients were <2. Values in recipients are consistent with ^1^H-NMR studies [37,38]. Almost all biomarkers found within embryos were predictive only at birth. In contrast, plasma biomarkers were found for every pregnancy endpoint in AV plus Holstein (9, 6, and 7 biomarkers predicted at Day-40, Day-62, and birth, respectively). Of these, only two biomarkers (the acids stearic and palmitic, in AV) showed clear significance at birth, either in the univariate study or the biomarker discovery study or both, while the remaining birth biomarkers (including all identified in Holstein) showed tendencies. Factors that govern early pregnancy in cattle depend mainly on recipients, whose pregnancy-predictive ability declines after Day-60 [8], and includes the critical peri-implantation period (Day-14 to Day-28) with the abundant embryo losses that hallmark recipient fertility [36]. These findings are in agreement with the predictive power and timing of biomarkers found in this study.

### 3.4. New Biomarker Strategies

In this study, we divided the recipient cohort by breeds, as suggested by discriminant analyses and previous findings with ^1^H-NMR [37,38]. Subdividing the datasets into controllable, non-random blocks improved the validation of predictions [37,38]. Accordingly, the set of biomarkers obtained were completely different between AV and Holstein recipients. In contrast, we discovered the biomarkers within the entire embryo dataset and found biomarker candidates valuable for “bovine embryos” notwithstanding oocyte origins, individual bulls, and breed bulls. We consider the combined predictive value of the F1 score as the most important finding in our study. When using biomarkers from two sides (i.e., embryo and recipient) shown in most cases, there is more predictive power at birth than the use of each biomarker as a single predictor. This was accompanied by improved statistical significance and predictive power within the F1 score, especially with the use of embryonic stages (only possible within the Holstein cohort), in line with the metabolic differences found within embryonic stages by other authors [80,81,82,83].

The F1 score also identified “hidden” biomarkers, which were not clear as single biomarkers (i.e., with non-significant *p* values), demonstrating the effect of the complementary confounders when compared to working only with either embryos or recipients ([8], this study). His is the case of heptadecanoic acid and citric acid. At the same time, the F1 score may change the value of the single biomarkers identified, e.g., stearic acid did not yield higher F1 scores than capric acid and glyceryl monostearate, despite the better value of stearic acid as a single biomarker. Within the validation procedure with the F1 score, the embryonic stage permitted considerable improvement in predictive ROC-AUC and significance, with combinations of capric acid and glyceryl-monostearate (embryonic) and hydrocinnamic acid, citric acid, and hippuric acid (recipient) being significant. The impact of the lipid metabolism in long-term survival made capric acid and glyceryl monostearate the best candidates to predict pregnancy to term, both individually and combined with plasma metabolites within F1 scores. Curiously, embryo CM metabolites failed to predict pregnancy at Day-40 and the only discovered candidate on Day-62 and birth (stearic acid) was not validated. In contrast, within Holstein recipients, candidates were more robust on Day-40 (*p* < 0.05 values and ROC-AUC > 0.880) than at birth (*p* tendencies and ROC-AUC < 0.800), while, within AV, all pregnancy endpoints were balanced in biomarker amounts.

We have identified candidate metabolite biomarkers that predict pregnancy to term with V/W embryos both in the embryo and the recipient. Within Holsteins, hydrocinnamic acid, alanine, and lysine predicted birth with ROC-AUC > 0.778. Beef recipients that reached birth showed lower concentrations of 6-methyl-5-hepten-2-one, stearic acid, palmitic acid, and hippuric acid (ROC-AUC > 0.832). Embryonal capric acid and glyceryl-monostearate gave the highest F1 scores (generally > 0.900) with plasma metabolites found both regulated (e.g., hippuric acid, hydrocinnamic acid) and, interestingly, not regulated (e.g., heptadecanoic acid, citric acid). Within embryos, the procedure is completely non-invasive, which likely permits correct classification of viable embryos without false negatives. Within embryos and recipients, the F1 score acts as a valuable tool that avoids confounders to obscure the biomarker value of candidate metabolites. The predictive values of individual biomarkers can be re-calculated by means of inferences from the complementary biomarkers (i.e., from the recipient or from the embryo). Such a procedure will be accomplished once more abundant and accurate information from metabolites under different real scenarios is available. The F1 score is here described for first time in mammalian pregnancy, allowing significant predictions at birth, which potentially includes discarding embryos that produce miscarriage, which is a damaging and costly event in breeding. We know that biomarker selection based on Metabolomics is not a realistic choice for on-field purposes. We envision electrochemical procedures working with a very low sample volume, portable or not, and specific laboratory ELISA or enzyme-based methods for targeted biomarkers. These assays take minutes to hours and return confident and repeatable results. Selection of embryos and recipients before ET with metabolite biomarkers can be reliable, and also cost-effective, especially if high genetic merit embryos are used. Such elite embryos arise from genomics programs, which may select superior, constitutive fertile recipients [36], but cannot select a specific cycle in which a recipient will become pregnant. The use of recipients and embryos with a well-profiled viability expectation will increase pregnancy rates, and reduce the need for recipients, which will be profitable for the breeding industry.

The metabolites discovered reveal a significant influence of lipid metabolism both on the embryo and the recipient, with a probable need for balance in fatty acids to allow pregnancy to proceed to term. The nutritional effects, manifested within the transformation of dietary phenolics, may include the microbiota and subsequent metabolism in the liver as important factors in the selection of cattle recipients.

## 4. Materials and Methods

**Figure 4:**
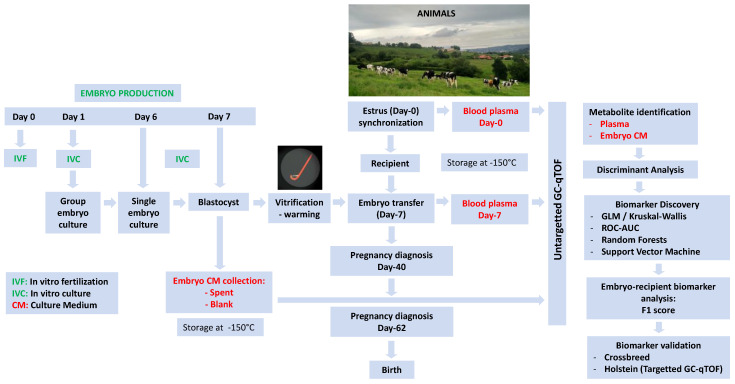
Experimental workflow.

All reagents were purchased from SIGMA (Madrid, Spain) unless otherwise stated. Experimental workflow is described in Figure 4.

### 4.1. Rationale

This study analyzed the metabolomic profiles in Day-0 and Day-7 recipient plasma samples and in embryo CM. V/W embryos were transferred and recipients diagnosed as pregnant or open (i.e., non-pregnant) at specific time endpoints (Day-40, Day-62, and birth). Metabolites in CM and plasma were analyzed as a function to predict pregnancy at each pregnancy endpoint.

Biomarker discovery must be guided by principles of population science to identify the biomarkers that represent the population under scrutiny, and not only the samples studied [4,84]. Thus, discovery of recipient biomarkers for pregnancy involves using IVP embryos and recipients from varied genotypes as a way to introduce random variability in both factors determining pregnancy (i.e., embryos and recipients). Variability in embryos means including random sources, such as different bulls, oocytes from different mothers, and different embryo production systems. However, it is not feasible to adapt each biomarker to the enormous existing assortment of laboratory culture conditions. Otherwise, the risk of dependence between biomarkers and a particular embryo production system would increase [37,38]. Ideally, biomarkers that behave as predictive in highly variable populations are more valuable. However, biomarker discovery can be approached as a block strategy within non-random factors, such as recipient breed and embryo cryopreservation [37,38], which can be controlled in the laboratory. Thus, a randomized design may entail more precision within each fixed condition (i.e., block) that can be predetermined.

### 4.2. In Vitro Embryo Production

Oocytes were aspirated from ovaries of slaughtered cows (Matadero de Guarnizo, Cantabria, Spain). Oocytes are contained in antral follicles visible in the ovarian surface. Each punctured follicle normally renders one oocyte surrounded by cumulus cells. Such a complex must undergo a process termed in vitro maturation (IVM) by which the oocyte becomes competent to be fertilized. The procedures for ovary collection, transportation, and IVM have been recently described in detail [3]. Matured oocytes were fertilized with commercial frozen sperm from *N* = 10 bulls with proven fertility, which was thawed and used for in vitro fertilization (IVF, Day 0). Motile sperm were obtained by a swim-up procedure. Spermatozoa processing and IVF procedures were described [85]. Briefly, sperm were incubated with pre-equilibrated Sperm-TALP (Tyrode’s albumin lactate pyruvate) for 1 h. The supernatant with motile sperm was recovered and centrifuged for 7 min at 200× *g*, leaving a spermatozoa pellet. IVF co-culture was performed in a fertilization medium (Fert-TALP) with heparin (10 μg/mL, Calbiochem, La Jolla, CA, USA). Spermatozoa were added at a concentration of 2 × 10^6^ cells/mL in 500-μL medium per well with a maximum of 50 cumulus-oocyte complexes. IVF was accomplished by incubating oocytes and sperm cells together for 18 to 20 h at 38.7 °C in 5% CO_2_ atmosphere with high humidity.

Fertilized oocytes were freed from cumulus cells by vortex and cultured in modified synthetic oviduct fluid (mSOF) containing amino acids (BME amino acids solution, 45 μL/mL, and MEM non-essential amino acids solution, 3.3 μL/mL), citrate (0.1 μg/mL), myo-inositol (0.5 μg/mL), and bovine serum albumin (BSA) (6 mg/mL) with or without 0.1% fetal calf serum (FCS). In vitro culture (IVC) was carried out at 38.7 °C, 5% CO_2_, 5% O_2_, 90% N_2_, and saturated humidity. During IVC, embryos progress in development until reaching the blastocyst stage. In vitro, the expanded blastocyst stage is preferred for ET because of its maximal likelihood of pregnancy both as fresh and after cryopreservation. In this study, embryos were cultured in groups until Day-6. On Day-6 (143 h after the onset of fertilization), excellent and good quality (grade 1 and grade 2) morulae and early blastocysts were selected and cultured individually in 12 µL mSOF with 0.5 mg/mL polyvinyl-alcohol PVA (P8136, replacing protein supplements) under mineral oil [44]. Blastocyst development was assessed on Day-7 (168 h after fertilization), at which time expanding and fully expanded blastocysts were selected for vitrification. Embryonic stages on Day-6 and Day-7 were recorded. Readers interested in blastocyst development rates, survival to cryopreservation, differential cell counts, and lipid contents are encouraged to read Murillo et al. [44]. For pregnancy and birth rates of these embryos transferred fresh and frozen/thawed, and birth weights, please go to Gómez et al. [3]. 

### 4.3. Embryo Vitrification and Warming

Vitrification is a procedure of cryopreservation allowing embryos to remain practically unaltered for years, facilitating logistics for ET. The vitrification procedures used in this study have been described in detail [86]. Briefly, expanded blastocysts were vitrified in two steps with fibre plugs (CryoLogic Vitrification Method, CVM). Procedures were performed on a heated surface (41 °C) in a warm room (25 °C). Embryos were handled with a basic vitrification medium (VM: TCM 199-HEPES + 20% (*v*/*v*) FCS). Each blastocyst was exposed to VM with 7.5% ethylene-glycol (EG, 102466-M), 7.5% DMSO (D2650, vitrification solution-1) for 3 min, and then moved into a droplet containing VM with 16.5% EG, 16.5% DMSO, and 0.5 M sucrose (vitrification solution-2, VS2). The time spent by the embryos in VS2 (including loading) was 20 to 25 s. Samples were vitrified by touching the surface of a supercooled block placed in LN2 with a hook. Vitrified embryos held in fibre plugs were stored in closed straws in LN2 until warming, which is a necessary step to revive the embryo prior to ET. The embryos were warmed in one step by directly immersing the fibre plug end in 800 L of 0.25 M sucrose in VM, where the embryo was kept for 5 min and washed twice in VM and twice in mSOF containing 6 mg/mL BSA and 10% FCS prior to ET. Embryo morphology was evaluated prior to ET, and embryos with large fragmentation or a degenerated appearance were discarded. 

### 4.4. Embryo Transfer and Pregnancy Diagnosis

Embryos were transferred in controlled conditions (experimental herd). For more detailed information on ET procedures and recipient traits, feeding, nutrition, and management, the readers are referred to our published work [37,38,41]. Recipient heifers from Holstein (1.84 average years old at the time of the first ET), Asturiana de los Valles (AV, 1.74 years) breeds and their crosses (1.76 years) were used. Holsteins and AV differ not only as dairy and beef breeds, respectively, but because the muscular hypertrophy (mh) phenotype present in AV cattle [87,88] is absent in Holsteins. The age/development stage of the embryo and the estrous cycle of the recipient need to be synchronized for a successful ET. Recipients were synchronized in their oestrus cycle with an intra-vaginal progestagen device (PRID Alpha, Ceva Salud Animal, Barcelona, Spain) for 10–11 days combined with a prostaglandin F2-alpha analogue (PG, Dynolitic, Leonvet, Leon, Spain) injected 48 h before progestagen removal. Recipients selected for transfer showed either standing estrus or, in the absence of estrous signs, Day-7 progesterone (P4) values > 3.5 ng/mL, P4 fold changes Day-7/Day-0 > 8, and a healthy corpus luteum monitored by ultrasonography prior to ET.

ETs were performed with V/W Day-7 expanded blastocysts. Embryos were non-surgically transferred to recipients under epidural anesthesia. Pregnancy was diagnosed by ultrasonography on Day 40 and on Day 62. Birth rates were monitored. Supplemental Table S6 shows all ETs performed in this study and their pregnancy rates.

### 4.5. Sample Collection for Metabolomics and Progesterone Analysis

Samples from embryo CM and recipient blood plasmas were collected for metabolomic analysis, always prior to the time of ET. The procedures have been described [6] with slight modifications. At the time of embryo removal from culture for vitrification on Day-7, spent CM (10 µL) and blank controls (i.e., droplets incubated without embryos) were collected, snap-frozen in LN_2_, and stored at −150 °C until metabolomic analysis. 

Blood plasma samples from recipients were taken from coccygeal vein puncture and collected into ethylenediaminetetraacetic acid (EDTA) vacuum tubes. Blood samples were taken at fixed times on Day-0 (i.e., 96 h after PG application, 48 h after progestagen removal) and Day-7 (216 h after progestagen removal, 5 h before the ET time). Blood tubes were immediately refrigerated at 4 °C, and centrifuged at 2000× *g* within 30 min of recovery. Supernatant plasma was aliquoted and stored at −150 °C until analysis.

Blood plasma P4 was measured on Day 0 and Day 7 by an enzyme-linked immunosorbent assay (ELISA) test (DRG^®®^ Progesterone ELISA, EIA-1561, DRG Diagnostics, Marburg, Germany). The test was sensitive starting from 0.5 ng/mL, and cross-reactivity from steroids other than P4 was less than 1%. Intra-assay and inter-assay coefficients of variation were 6% and 7%, respectively.

### 4.6. Metabolomic Analysis of Plasma and Embryo Culture Media

For metabolomic analysis in the discovery study, plasma samples of study subjects and the embryo CM were analyzed by untargeted metabolomic analyses. A GC-qTOF platform was used to cover a major number of interesting metabolites. Metabolite extraction was performed by the protein precipitation method, adding eight volumes of methanol:water (8:2 *v*/*v*) containing an internal standard mixture (succinic acid-d4, myristic acid-d27, glicerol-13C3, and D-glucose-13C6) to 100 µL of serum or 10 µL of CM. Then the samples were mixed and incubated at 4 °C for 10 min, centrifuged at 15,000 rpm, and the supernatant was evaporated to dryness and freeze dried before metabolite derivatization (metoximation and silylation). The derivatized compounds were analyzed by GC-qTOF (model 7200 of Agilent, Santa Clara, CA, USA) using the metabolomic Fiehn Method with a J&W Scientific HP5-MS (30 m × 0.25 mm, i.d., 0.25-μm film capillary column) and helium as the carrier gas under an oven program from 60 to 325 °C. Ionization was done by electronic impact (EI) with electron energy of 70 eV and operated in a full scan mode. Raw data were first examined using Unknown Analysis software from Agilent using metabolomic Fiehn library (from Agilent), which contains more than 1.400 metabolites for the screening by matching their EI mass spectrum and retention time. After putative identification of metabolites, these were semi-quantified using the internal standard (IS) response ratio across all samples. The IS ratio was calculated by the quotient between the area under the chromatographic peak of each identified metabolite and the area of their assigned IS. It is also dimensionless. This proportion is assumed to be concentration-dependent for exploratory metabolomic studies. The IS was assigned to each of the identified compounds based on their chemical similarity.

For the validation metabolomics study, the experimental procedure was as explained before, except that analytical standards for selected metabolites were used for identity confirmation and absolute quantification with internal standard calibration curves.

### 4.7. Experimental Design and Statistics

In this work, recipient variability was ensured with cattle from different breeds (Holstein and AV) and batches of different animals used in a three-year period (discovery study) and a subsequent two-year period (validation study). No special conditions were required other than testing free of the most prevalent (*N* = 7) infectious diseases affecting reproduction with a potential incidence in the region, healthy after clinical examination and normal after gynecological examination. Variability in embryos was based on oocytes (blind ovary collection from abattoir) fertilized with different bulls (AV (*N* = 4), Holstein (*N* = 3)). The blood plasma of recipients was collected at fixed times on Day-0 and Day-7 (4–6 h before ET). After warming, all vitrified and thawed Day-7 embryos were transferred, and no embryo or ET was discarded by any condition not described here over the experimental course of five years (Discovery and Validation studies). Pregnancy was monitored on days 40, 62, and birth. Metabolomic analysis of embryo CM and plasma was conducted as shown above to obtain relative (discovery study and part of validation study) or absolute (validation study) metabolite concentrations.

Statistical analyses of metabolite concentrations were conducted using SAS/STAT (Version 9.2, SAS Institute Inc. Cary, CA, USA) and Metaboanalyst 4.0 (Alberta, Canada) [89].

#### 4.7.1. Analyzing Breed Effects in Embryos and Recipients

We first analyzed the influence on the metabolome of the major fixed factors’ embryo sire breed and recipient breed in the two sample collection groups (*N* = 36 from Day-7 and *N* = 35 from Day-0, as one sample from Day-0 was not collected). The embryo CM and the recipient plasma were examined with PLS-DA and sPLS-DA after data transformation with Pareto scaling (Metaboanalyst 4.0).

#### 4.7.2. Biomarker Studies

##### Identification of Candidate Metabolites

Subsequent discovery studies were followed by independent analysis of single metabolites (SAS/STAT). Normality in distribution of each metabolite was analyzed (Proc UNIVARIATE). Parametric (ANOVA) and non-parametric (Kruskal-Wallis [KW]) statistics were used, as some metabolite concentrations were not normally distributed. Within CM, the effects of sire breed and embryonic stage prior to (Day-6) and after (Day-7) individual culture were investigated for single metabolites. The embryo culture in the discovery study was performed in detail with SOF + BSA (*N* = 34 samples), and *N* = 2 samples produced in SOF + BSA + FCS that completed the *N* = 36 dataset, which had no influence at any level analyzed. Subsequently, we identified DM, in accordance with the status (pregnant, non-pregnant) diagnosed at each pregnancy endpoint (Day-40, Day-62, and birth). Metabolites with significant differences (*p* < 0.05) and Bonferroni correction (*p* < 0.05) as a false discovery rate (FDR) were considered biomarker candidates.

##### Identification of Biomarker Candidates

Metabolite biomarker candidates were analyzed in two ways:

1. Single biomarker identification: which included a univariate study with a Receiver Operator Characteristic—area under curve (ROC-AUC) (T-Test, *p* < 0.05, ROC-AUC > 0.650) within selected metabolites at each pregnancy endpoint in each sample subset analyzed, i.e., embryo CM and plasmas (Metaboanalyst 4.0). The effects of embryonic stages on Day-6 (i.e., morula and early blastocysts) as controllable variation sources were included in this study. Subsequently, a multivariate study with ROC curve-based exploratory analysis was carried out. The classification and feature ranking methods that showed best agreement with univariate studies were RF (Mean Decrease Accuracy –MDA- for CM metabolites) and SVM (Selected Frequency—SF—for plasma metabolites).
2. Combined biomarker identification: we analyzed the PA of multiple features with the largest ROC-AUC values within the embryo CM and recipient plasma. The “Multivariate ROC curve based exploratory analysis (Explorer)” tool from Metaboanalyst was used to test the algorithms SVM, PLS-DA, and RF. Subsequently, ROC-AUC values for multiple metabolite combinations were calculated using the “ROC curve-based model evaluation (Tester)” tool.
3. F1 score: The procedure was intended to collect information from the recipient and the embryo to potentially increase predictability. Each single biomarker identified in CM was combined with each metabolite identified in plasma to obtain an index (F1 score) that represents the aggregate predictive power of CM and plasma for each pregnancy specific endpoint. A Boolean matrix was performed for each metabolite in terms of allocation (1 = true) or not (0 = false) within the range of pregnant animals. F1 calculations were performed on the Boolean product vector of pairs of metabolites (i.e., 1 metabolite from CM and 1 metabolite from plasma/day/pregnancy endpoint) (False*False = False, True*False = True, False*True= True, True*True = True). For a visual representation, see a real example in Supplemental Table S4 (i.e., CM, its corresponding plasma relative concentration, and the combined F1 score). The probability value of the F1 score was calculated by a Chi-square test.

#### 4.7.3. Biomarker Validation

Validation studies were performed with independent samples of CM and plasma corresponding to V/W embryos transferred to different Holstein recipients (*N* = 19). 

## Figures and Tables

**Table 1:** Univariate and biomarker identification studies of candidate metabolites for pregnancy endpoints birth and day-62 in culture medium (CM) of embryos transferred with vitrified/warmed embryos (CM samples from *N* = 36 ETs).

	*p* Value		Pregnancy	Univariate Study			Biomarker Discovery Study				
Metabolite	BB ^1^	Day-6-E ^2^	Endpoint	*p*-Value	Open	Pregnant	*p*-Value	FCh	ROC-AUC	Open	Pregnant
Glyceryl monostearate	0.406	0.077	Birth	0.023	2454 ± 698	940 ± 468	**0.060**	2.829	0.670	11/17	11/18
Capric acid	0.364	0.742	Birth	0.048	329 ± 68	188 ± 45	0.021	4.472	0.728	10/17	15/19
Palmitic acid	0.507	0.341	Birth	**0.089**	17,719 ± 2992	12,460 ± 1978	**0.051**	5.381	0.669	12/17	12/19
Stearic acid	0.350	0.188	Birth	0.019	75,827 ± 9612	51,951 ± 6356	0.016	5.899	0.743	13/17	14/19
											
Stearic acid	0.350	0.188	D-62	0.023	75,692 ± 11,580	52,453 ± 6092	0.022	5.612	0.733	11/15	14/21

Tendencies 0.05 < *p* < 0.09 are in bold. *p* values from major effects are shown: bull breed (BB ^1^) and embryonic stage on Day-6 (Day-6-E ^2^). FCh: Log fold-change in concentrations (open/pregnant). *p* values: Kruskal-Wallis test (Glyceryl monostearate) or General Linear Model (GLM) procedure (Capric, Palmitic, and Stearic acids), Bonferroni correction (*p* < 0.05) (Univariate study) and *T*-test (Biomarker discovery study). In contrast, glyceryl monostearate (one sample deleted as out layer 1200× mean value), capric acid, and stearic acid concentrations were significantly lower in embryos that reached birth (*p* < 0.05), and palmitic acid tended to decrease (Table 1). None of those metabolites was affected by bull breed, but glyceryl monostearate concentrations tended to differ at Day-6 between morula and blastocyst stages (*p* = 0.077). ROC-AUC: Receiver Operator Characteristic—area under curve.

**Table 2:** Univariate analysis and biomarker identification studies (ROC-AUC) of candidate metabolites in plasma collected on Day-0 (*N* = 16) and Day-7 (*N* = 17) from Holstein recipients transferred with vitrified/warmed embryos and diagnosed at pregnancy endpoints Day 40, Day 62, and birth.

	Day	Pregnancy	Univariate Study			Biomarker Discovery Study				
Metabolite	Plasma	Endpoint	*p*	Open	Pregnant	*p*	FCh	ROC-AUC	Open	Pregnant
Hydrocinnamic acid	0	Birth	**0.059**	362 ± 34	255 ± 44	0.044	0.495	0.797	6/8	6/8
										
Hydrocinnamic acid	0	Day-40	0.012	400 ± 34	254 ± 26	0.005	0.666	0.900	5/6	8/10
2-hydroxybiphenyl	0	Day-40	0.013	271 ± 30	363 ± 23	0.031	−0.395	0.900	5/6	8/10
Glycerol-phosphate	0	Day-40	0.017	1315 ± 307	1401 ± 238	**0.062**	−0.586	0.883	6/6	8/10
										
Alanine	7	Birth	**0.065**	1662 ± 182	2163 ± 172	**0.065**	−0.357	0.792	6/8	6/9
Lysine	7	Birth	**0.067**	148 ± 25	223 ± 24	0.049	−0.497	0.778	6/8	7/9
										
Threonine	7	Day-62	**0.051**	93 ± 18	141 ± 15	**0.052**	−0.669	0.800	5/7	8/10

Open and pregnant: concentrations in arbitrary units (Univariate study) and samples correctly identified/total samples (Biomarker discovery study). *p* values: Kruskal-Wallis test or General Linear Model procedure with Bonferroni correction *p* < 0.05 (Univariate study) and *t*-test (Biomarker discovery study). Significant differences: *p* < 0.05. Tendency (bold) 0.05 < *p* <0.07. FCh = Log Fold change. ROC-AUC: Receiver Operator Characteristic—area under curve.

**Table 3:** Univariate and biomarker discovery study (ROC-AUC) of candidate metabolites at pregnancy endpoints (Day 40, Day 62, and birth) in Day-0 (*N* = 13) and Day-7 (*N* = 13) plasma of Asturiana de los Valles recipients transferred with vitrified/warmed embryos (data from Day-62 and Day-40 were the same).

	Day	Pregnancy	Univariate Study			Biomarker Discovery Study				
	Plasma	Endpoint	*p*	Open	Pregnant	*p*	FCh	ROC-AUC	Open	Pregnant
Stearic acid	0	Birth	0.022	3668 ± 96	3204 ± 104	0.007	0.191	0.881	6/7	6/6
Palmitic acid	0	Birth	0.007	2901 ± 215	2136 ± 221	0.013	0.367	0.976	6/7	6/6
6-methyl-5-hepten-2-one	0	Birth	**0.055**	108 ± 1.9	115 ± 2.0	**0.055**	−0.078	0.833	6/7	5/6
										
Palmitic acid	0	D40–D62	0.015	286 ± 17	223 ± 16	0.020	0.342	0.929	5/6	6/7
										
Hippuric acid	7	Birth	0.021	2385 ± 144	1663 ± 148	**0.071**	0.430	0.857	5/7	5/6
										
Hippuric acid	7	D40–D62	0.045	2458 ± 230	1839 ± 213	0.045	0.454	0.833	4/6	6/7
L-Valine	7	D40–D62	**0.056**	4254 ± 443	3607 ± 410	**0.056**	0.596	0.857	5/6	6/7
N-(2-hydroxyethyl) iminodiacetic acid 2	7	D40–D62	0.022	10,743 ± 492	9150 ± 455	0.037	0.230	0.881	5/6	7/7
Dehydroascorbic acid	7	D40–D62	0.034	1213 ± 66	1012 ± 49	0.034	0.302	0.857	5/6	6/7

Open and pregnant: concentrations in arbitrary units (Univariate study) and samples correctly identified/total samples (Biomarker discovery study). *p* values: Kruskal-Wallis test or GLM procedure with Bonferroni correction *p* < 0.05 (Univariate study) and *t*-test (Biomarker discovery study) significant differences: *p* < 0.05. Tendency (bold): 0.05 < *p* < 0.072. FCh = Log Fold change.

**Table 4:** Pairs of single biomarker metabolites in the embryo culture medium and plasma (Day-0 or Day-7) predict pregnancy at the indicated endpoints and within recipient breeds (AV: Asturiana de Valles, Holstein) by calculation of F1 scores within aggregate (Day-6 embryonic stages not considered) or B/M (Day-6 separate blastocyst and morula stages analyzed).

Breed	Embryo Culture Medium		Recipient Plasma			Endpoint	F1 Score		
	Metabolite	ROC-AUC	Metabolite	Day	ROC-AUC		Aggregate	B/M	*p*
AV	Capric acid	0.728	Palmitic acid	0	0.952	Birth	1.000		<0.001
AV	Capric acid	0.728	Stearic acid	0	0.881	Birth	1.000		<0.001
AV	Capric acid	0.728	Heptadecanoic acid	0	0.810	Birth	0.923		0.002
AV	Glyceryl-Monostearate	0.721	Palmitic acid	0	**0.952**	Birth	**0.923**		0.002
AV	Glyceryl-Monostearate	0.721	Stearic acid	0	0.881	Birth	0.923		0.002
AV	Glyceryl-Monostearate	0.721	Heptadecanoic acid	0	0.810	Birth	0.923		0.002
AV	Capric acid	0.728	L-Valine	7	0.762	Birth	0.900		0.008
AV	Capric acid	0.728	2-hydroxybiphenyl	7	0.714	Birth	0.923		0.002
AV	Capric acid	0.728	N-(2-hydroxyethyl) iminodiacetic acid 2	7	0.762	Birth	0.923		0.002
AV	Capric acid	0.728	Hippuric acid	7	0.857	Birth	0.857		0.008
AV	Capric acid	0.728	Dehydroascorbic acid	7	0.738	Birth	0.857		0.008
									
AV	Glyceryl-Monostearate	0.695	Palmitic acid	0	**0.904**	D62	**0.875**		0.008
AV	Glyceryl-Monostearate	0.695	Stearic acid	0	0.733	D62	0.875		0.008
AV	Glyceryl-Monostearate	0.695	Heptadecanoic acid	0	0.810	D62	0.875		0.008
									
AV	Capric acid	0.662	Stearic acid	0	0.785	D40	0.933		0.002
AV	Capric acid	0.662	Heptadecanoic acid	0	0.786	D40	1.000		<0.001
AV	Glyceryl-Monostearate	0.687	Palmitic acid	0	**0.904**	D40	**0.875**		0.008
AV	Glyceryl-Monostearate	0.687	Stearic acid	0	0.733	D40	0.875		0.008
AV	Glyceryl-Monostearate	0.687	Heptadecanoic acid	0	0.810	D40	0.933		0.002
									
Holstein	Capric acid	0.728	Hydrocinnamic acid	0	0.797	Birth	0.889	0.933	<0.001
Holstein	Capric acid	0.728	Glycerol-phosphate	0	0.625	Birth	0.842	0.933	<0.001
Holstein	Capric acid	0.728	Citric acid	0	0.625	Birth	0.889	0.941	<0.001
Holstein	Glyceryl-Monostearate	0.695	Citric acid	0	0.625	Birth	0.842	0.889	0.002
Holstein	Capric acid	0.728	L-Alanine	7	0.792	Birth	0.900	0.900	0.001
Holstein	Capric acid	0.728	L-Threonine	7	0.764	Birth	0.900	0.947	<0.001
Holstein	Capric acid	0.728	Hydrocinnamic acid	7	0.583	Birth	0.900	0.947	<0.001
Holstein	Capric acid	0.728	Glycerol-phosphate	7	0.652	Birth	0.900	0.947	<0.001
									
Holstein	Glyceryl-Monostearate	0.695	Hydrocinnamic acid	0	0.794	D62	0.857	0.903	0.001
									
Holstein	Capric acid	0.670	Hydrocinnamic acid	0	0.900	D40	0.952	0.952	<0.001
Holstein	Glyceryl-Monostearate	0.687	Hydrocinnamic acid	0	0.900	D40	0.909	0.952	<0.001
Holstein	Capric acid	0.670	L-Valine	7	0.606	D40	0.917	0.917	0.002
Holstein	Capric acid	0.670	L-Threonine	7	0.757	D40	0.917	0.957	<0.001
Holstein	Glyceryl-Monostearate	0.687	L-Threonine	7	0.757	D40	0.917	0.917	0.002

F1 scores that do not show higher values than ROC-AUC values from single metabolites are in bold.

**Table 5:** Targeted validation (GC-MS) at birth by F1 score (with or without separation of blastocyst and morula as Day-6 embryonic stages) of metabolite pairs selected in embryo culture medium (*N* = 19) and Holstein recipient Day-0 plasma (*N* = 19).

Combined Metabolites		With Day-6 Embryonic Stage			Without Day-6 Embryonic Stage		
Culture Medium	Plasma	True	F1 Score	*p* Value	True	F1 Score	*p* Value
Capric acid	Hydrocinnamic acid	14/19	0.848	0.013	ND		
Glyceryl-monostearate	Hydrocinnamic acid	15/19	0.882	0.005	14/19	0.848	0.013
Capric acid	Citric acid	16/19	0.889	0.002	ND		
Glyceryl-monostearate	Citric acid	16/19	0.914	0.002	ND		
Capric acid	Hippuric acid	13/19	0.813	0.033	ND		
Glyceryl-monostearate	Hippuric acid	15/19	0.882	0.005	14/19	0.848	0.013

ND: Not determined because less true values were identified than any or both metabolites individually. True: samples correctly identified as pregnant (true positives) + open (true negatives)/total samples analyzed. *p* value analyzed by Chi-Square Mantel-Haenszel.

## Data Availability

The data presented in this study are available on reasonable request from the corresponding author. The data are not publicly available due to privacy and/or ethical concerns.

## Supplementary Materials

The following are available online at https://www.mdpi.com/2218-1989/11/3/162/s1. Supplemental Figure S1. Partial least square discriminant analysis (PLS-DA) (1a, 1c, 1e) and Sparse-partial least square discriminant analysis (sPLS-DA) (1b, 1d, 1f) of the metabolome obtained from embryo culture medium (1a, 1b), Day-7 recipient plasma (1c, 1d), and Day-0 recipient plasma (1e, 1f). Comparisons performed were between embryos sired by Asturiana de los Valles (1) and Holstein (0) bulls, and recipients belonging to the same breeds. Close to significant separation (empirical *p*-values) is appreciated within Day-0 plasma samples by 100 permutation analysis for PLS-DA (*p* < 0.10) (1d), and appreciable separation is noted within sPLS-DA (1f). In contrast, PLS-DA analysis showed that CM (*p* = 0.37) and Day-7 plasma (*p* = 0.17) clustered together. Supplemental Figure S2. Predictive accuracy plots representative of combinations of two, three, or four features obtained using Partial Least Square—Discriminant Analysis (PLS-DA 1.1), Random Forests (RF 1.2), and Support Vector Machine (SVM 1.3). Red dots indicate the highest predictive mean accuracy obtained with each model, which corresponds to combinations of two metabolites for PLS-DA (1.1) and RF (1.2), and three metabolites for SVM (1.3). The highest mean predictive value was given by PLSDA and two metabolites (X1). Supplemental Figure S3. ROC-AUC values for specific combinations of two metabolites calculated by Partial Least Square-Discriminant analysis based on predictive accuracy plots shown in Supplemental Figure S2. 3.a: Capric acid + Stearic acid. 3.b: Capric acid + Palmitic acid. 3.c: Capric acid + Glyceryl monostearate. 3.d: Glyceryl monostearate + Palmitic acid. 3.e: Glyceryl monostearate + Stearic acid. 3.f: Stearic acid + Palmitic acid (95% confidence interval values are depicted). Supplemental Table S1. Metabolites identified in embryo culture medium. Supplemental Table S2. Metabolites identified in recipient plasma both on Day-0 and Day-7. Supplemental Table S3. Individual ETs performed and description of recipients, embryos, bull breed, and pregnancy diagnosis to term in discovery study, and validation study with Holstein recipients. Supplemental Table S4. Capacity of candidate metabolite biomarkers identified in embryo culture medium (CM) and Day-0 recipient plasma to predict pregnancy to term in Asturiana de los Valles recipients as single biomarkers and combined within an F1 score. Supplemental Table S5. Relative concentrations by targeted GC-MS (with separation of blastocyst and morula as Day-6 embryonic stages) of metabolites selected in embryo culture medium (CM) containing bovine serum albumin (BSA) or fetal calf serum (FCS) + BSA subtracted from blanks (CM with no embryos), measured in *N* = 19 samples corresponding to embryos transferred to Holstein recipients that differed in reaching pregnancy to term or not. Supplemental Table S6. Embryo transfers with vitrified/warmed embryos and recipient breeds used in experiments and their pregnancy rates on Day-40, Day 62, and birth.
