# Supplementary material for: Metabolites Secreted by Bovine Embryos In Vitro Predict Pregnancies That the Recipient Plasma Metabolome Cannot, and Vice Versa
Source: Metabolites. 2021 Mar 11;11(3):162. doi: 10.3390/metabo11030162 (PMC7999939; doi:10.3390/metabo11030162)

Supplemental Figure 1

Partial least square discriminant analysis (PLS-DA) (1a, 1c, 1e) and Sparse-partial least square discriminant analysis (sPLS-DA) (1b, 1d, 1f) of the metabolome obtained from embryo culture medium (1a, 1b), Day-7 recipient plasma (1c, 1d), and Day-0 recipient plasma (1e, 1f). Comparisons performed were between embryos sired by Asturiana de los Valles (1) and Holstein (0) bulls, and recipients belonging to the same breeds. Close to significant separation (empirical p-values) is appreciated within Day-0 plasma samples by 100 permutation analysis for PLS-DA (P<0.10) (1d), and appreciable separation is noted within sPLS-DA (1f). In contrast, PLS-DA analysis showed that CM (P=0.37) and Day-7 plasma (P=0.17) clustered together.

Supplemental Figure 1

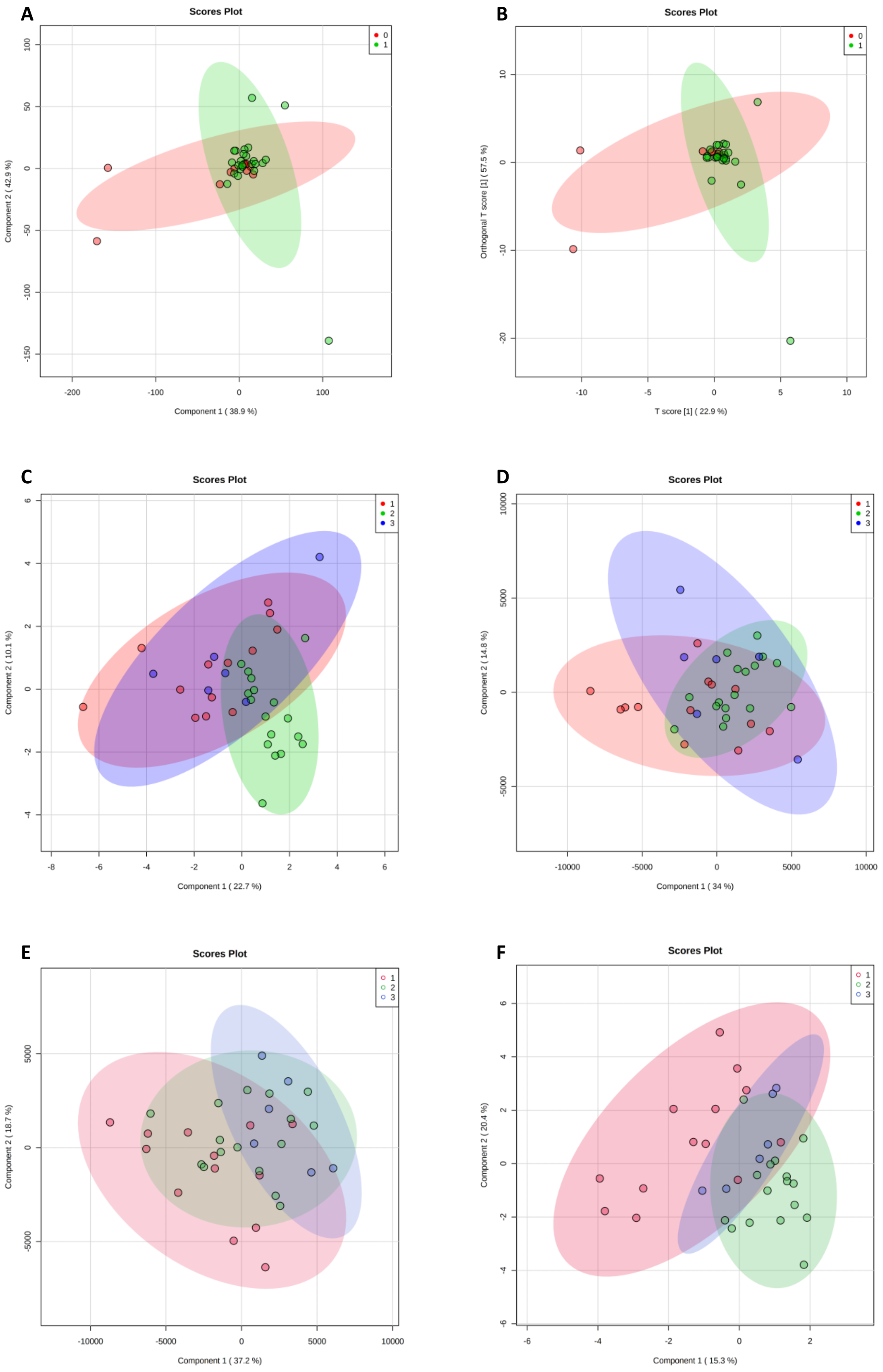

Supplemental Figure 2

Predictive accuracy plots representative of combinations of 2, 3 or 4 features obtained using Partial Least Square – Discriminant Analysis (PLS-DA 1.1), Random Forests (RF 1.2) and Support Vector Machine (SVM 1.3). Red dots indicate the highest predictive mean accuracy obtained with each model, that corresponds to combinations of 2 metabolites for PLS-DA (1.1) and RF (1.2), and 3 metabolites for SVM (1.3). The highest mean predictive value was given by PLSDA and 2 metabolites (X1).

Suppl Fig 2

## 2.1: Predictive accuracies by PLS-DA

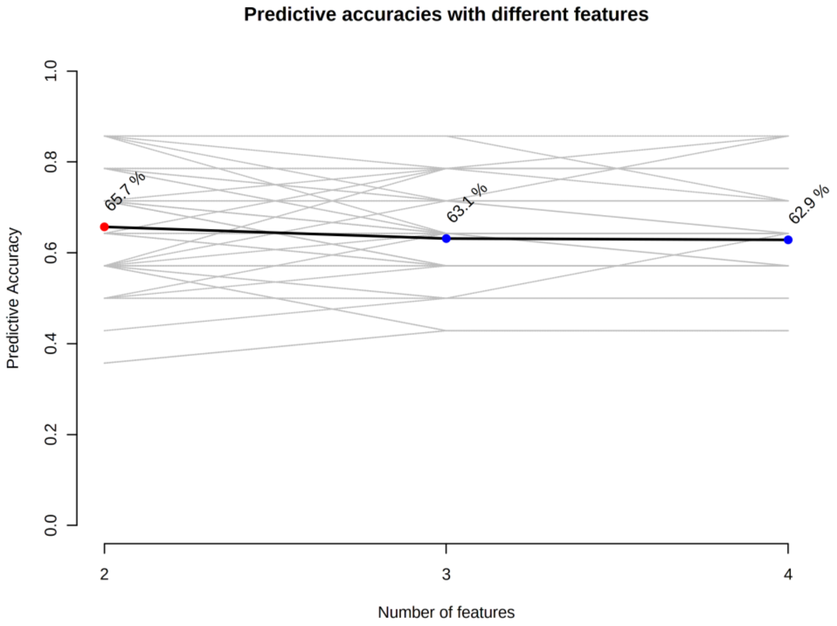


## 2.2: Predictive accuracies by Random Forests

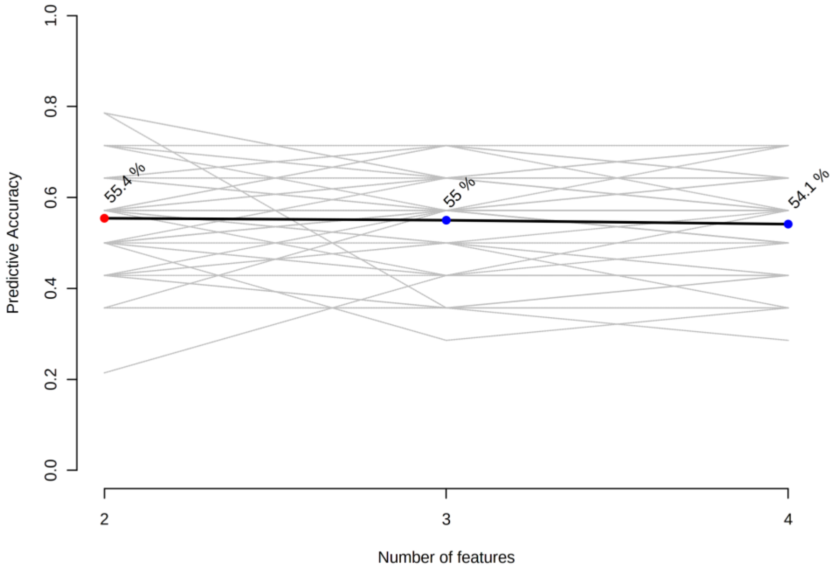


## 2.3: Predictive accuracies by Support Vector Machine

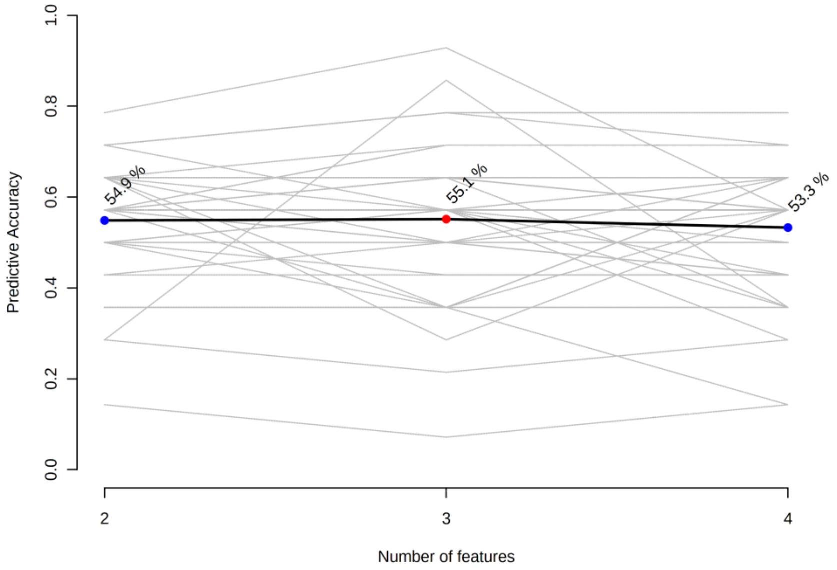

**Supplemental Figure 3**

ROC-AUC values for specific combinations of 2 metabolites calculated by Partial Least Square-Discriminant analysis based on predictive accuracy plots shown in Suppl Fig 2.
3.1: Capric acid + Stearic acid; 3.2: Capric acid + Palmitic acid; 3.3: Capric acid + Glyceryl monoestearate; 3.4: Glyceryl monoestearate + Palmitic acid; 3.5: Glyceryl monestearate + Stearic acid; 3.6: Stearic acid + Palmitic acid (95% confidence interval values are depicted).

Suppl Fig 3

3.1: Capric acid + Stearic acid

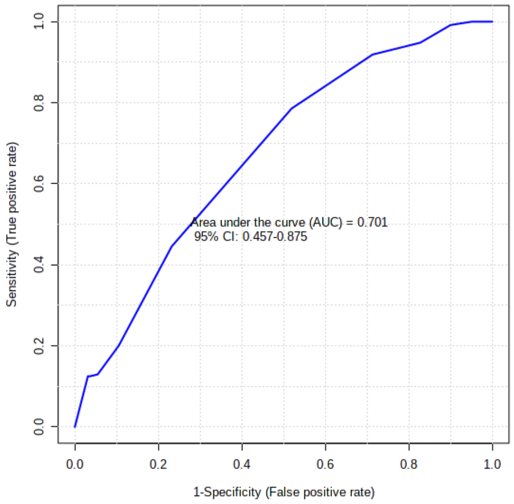


3.2: Capric acid + Palmitic acid

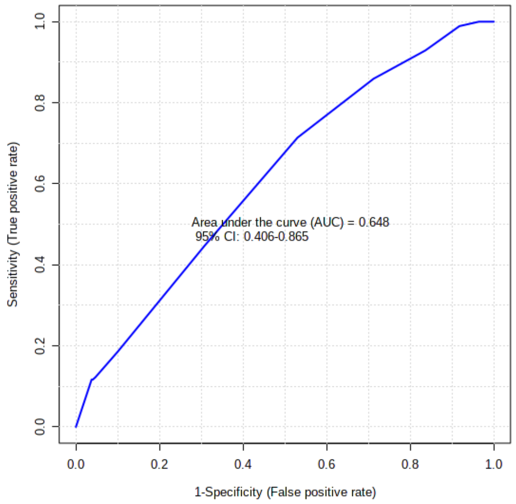


3.1: Capric acid + Glyceryl monoestearate

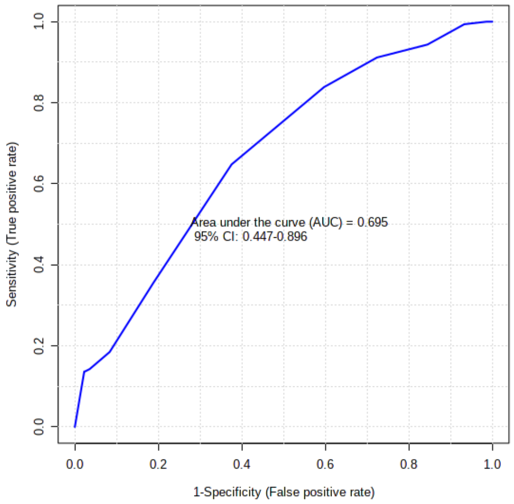


3.4: Glyceryl monoestearate + Palmitic acid

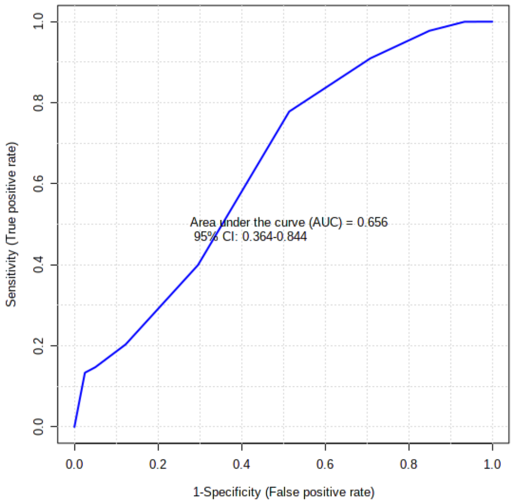


3.5: Glyceryl monoestearate + Stearic acid

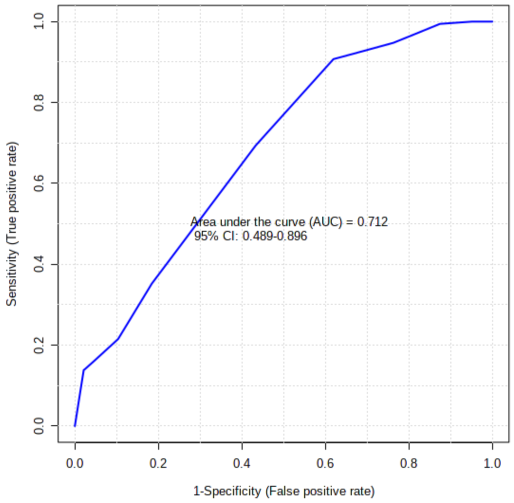


3.6: Stearic acid + Palmitic acid

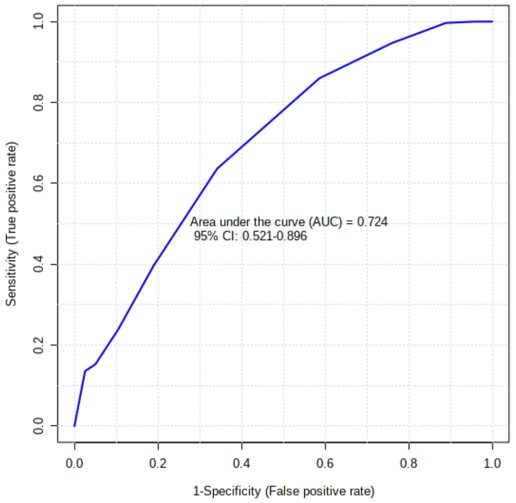

Supplement: Supplementary file 1 [file metabolites-11-00162-s001.zip › SUPPLEMENTAL FIGURES 240121.pdf]
