# Supplementary material for: Metabolites Secreted by Bovine Embryos In Vitro Predict Pregnancies That the Recipient Plasma Metabolome Cannot, and Vice Versa
Source: Metabolites. 2021 Mar 11;11(3):162. doi: 10.3390/metabo11030162 (PMC7999939; doi:10.3390/metabo11030162)
Supplement: Supplementary file 1 [file metabolites-11-00162-s001.zip › SUPPLEMENTAL TABLES 260221.pdf]

Supplemental Table S1

Metabolites identified in embryo culture medium

Metabolite*	Mass (m/z)**	RT (min)	CAS Number
2-hydroxypyridine	152.05	5.48	142-08-5
Pyruvic acid	174.11	5.75	127-17-3
L-(+) lactic acid	147.07	5.91	79-33-4
L-valine 1	72.08	6.24	72-18-4
Acetohydroxamic acid	146.06	6.64	546-88-3
3-hydroxypyridine	152.05	6.94	109-00-2
Oxalic acid	147.07	7.06	144-62-7
L-leucine 1	86.10	7.22	61-90-5
DL-isoleucine 1	86.10	7.52	443-79-8
2-ketoisocaproic acid 2	200.07	8.10	4502-00-5
Ethanolamine	73.05	8.36	141-43-5
L-ornithine 2	144.12	8.47	6600-40-4
Benzoic acid	179.05	8.50	65-85-0
L-serine 1	116.05	8.70	56-45-1
Caprylic acid	201.13	8.72	124-07-2
Phosphoric acid	299.07	8.98	7664-38-2
Glycerol	147.07	8.99	56-81-5
L-threonine 1	73.05	9.22	72-19-5
Glycine	174.11	9.40	56-40-6
Succinic acid	148.07	9.41	29915-38-6
Glyoxylic acid	184.04	9.76	298-12-4
Pelargonic acid (nonanoic acid)	215.09	10.03	112-05-0
Capric acid	229.07	11.29	334-48-5

L-glutamic acid 3 (dehydrated)	156.01	12.20	56-86-0
Aspartic acid 2	232.12	12.22	56-84-8
Phenylalanine 1	120.06	12.44	63-91-2
Lauric acid	257.07	13.63	143-07-7
N-(2-hydroxyethyl)iminodiacetic acid 2	312.40	15.56	93-62-9
Citric acid	273.10	15.70	5949-29-1
Myristic acid	285.05	15.76	544-63-8
Tyrosine 1	179.05	16.25	60-18-4
L-gulonic acid gamma-lactone	333.06	16.51	1128-23-0
Gluconic acid lactone 2	217.11	16.67	90-80-2
Palmitic acid	313.40	17.72	64519-82-0
Myo-inositol	305.14	18.47	87-89-8
Stearic acid	117.08	19.51	57-11-4
1 monostearin	400.01	23.82	123-94-4

*1 and 2 indicates different number TMS derivative moieties

**Quantifier ion used (identification was based on full EI spectrum match)

Supplemental Table S2

Metabolites identified in recipient plasma both on Day-0 and Day-7

Metabolite*	Mass (m/z)**	RT (min)	CAS Number
L-(+) lactic acid	235.12	5.01	79-33-4
Pyruvic acid	174.12	5.75	127-17-3
Glycolic acid	147.07	6.11	79-14-1
L-alanine 1	73.05	6.49	56-41-7
Ornithine 1	75.03	6.55	6600-40-4
Acetohydroxamic acid	133.02	6.64	546-88-3
6-methyl-5-hepten-2-one	240.24	6.75	110-93-0
Oxalic acid	147.07	6.93	144-62-7
2-hydroxybutyric acid	133.02	7.05	565-70-8
Propylamine 1	232.13	7.07	2043-43-8
2-ketoisocaproic acid 2	200.07	8.10	4502-00-5
L-valine 2	144.12	8.13	72-18-4
2-butyne-1,4-diol	147.07	8.30	110-65-6
ethanolamine	174.12	8.36	141-43-5
Benzoic acid	179.12	8.49	65-85-0
Urea	189.09	8.51	57-13-6
L-serine 1	116.09	8.69	56-45-1
L-leucine 2	158.07	8.92	61-90-5
DL-isoleucine 2	158.07	9.23	443-79-8
Glycine	174.12	9.40	56-40-6
Glyoxylic acid	184.04	9.77	298-12-4
Fumaric acid	245.12	9.88	17013-01-3
L-threonine 2	219.11	10.54	72-19-5

Hydrocinnamic acid	104.06	10.79	501-52-0
DL-3-aminoisobutyric acid 2	174.12	11.35	10569-71-9
Trans-4-hydroxy-L-proline 2	230.11	12.29	51-35-4
Iminodiacetic acid 2	232.13	12.31	142-73-4
Phenylalanine 1	120.06	12.44	63-91-2
Creatinine	115.00	12.65	60-27-5
Threonic acid	292.13	12.80	7306-96-9
Alpha ketoglutaric acid	198.08	12.87	328-50-7
2-hydroxybiphenyl	211.00	13.11	90-43-7
L-glutamic acid 2	246.14	13.39	56-86-0
2-amino-2-methyl-1,3-propanediol 2	192.09	13.46	115-69-5
Lauric acid	257.07	13.63	143-07-7
Pyrophosphate	451.08	14.02	0-00-0
Trans-aconitic acid	229.11	14.83	585-84-2
Glycerol 1-phosphate	357.07	15.10	34363-28-5
Azelaic acid	317.10	15.27	123-99-9
N-(2-hydroxyethyl)iminodiacetic acid 2	120.06	15.56	93-62-9
L-ornithine 2	142.07	15.62	70-26-8
Citric acid	274.10	15.70	5949-29-1
Hippuric acid 2	206.11	15.72	495-69-2
Myristic acid	285.04	15.76	544-63-8
D-allose 2	157.07	16.02	579-36-2
1,5-anhydro-D-sorbitol	217.11	16.04	154-58-5
D-mannose 1	157.07	16.10	87-78-5
phenaceturic acid 2	91.05	16.16	500-98-1
dehydroascorbic acid 3	157.07	16.45	490-83-5

D (+) galactose 1	205.11	16.54	59-23-4
D-Glucose	319.15	16.62	59-23-4
L-lysine 2	174.12	16.68	56-87-1
D (+)altrose 1	273.10	16.76	1990-29-0
Tyrosine 2	218.11	16.85	60-18-4
Galacturonic acid 1	333.06	17.03	14982-50-4
3-indoleacetic acid	202.11	17.06	87-51-4
Conduritol epoxide 2	217.11	17.28	6090-95-5
Isopropyl beta-D-glucoside	217.11	17.38	367-93-1
Palmitoleic acid	311.28	17.53	373-49-9
Palmitic acid	313.26	17.72	64519-82-0
Allantoin 3	259.12	18.18	97-59-6
Myo-inositol	305.14	18.47	87-89-8
Heptadecanoic acid	117.04	18.63	506-12-7
Linoleic acid	67.06	19.25	60-33-3
Oleic acid	339.04	19.29	112-80-1
L-tryptophan 2	202.11	19.49	73-22-3
Stearic acid	341.02	19.51	57-11-4
1-stearoyl-rac-glycerol	399.33	23.82	123-94-4
Alpha tocophereol	237.11	26.30	10191-41-0
Cholesterol	329.32	26.43	57-88-5
5alpha-Cholestanol	215.12	26.49	80-97-7

*1 and 2 indicates different number TMS derivative moieties

**Quantifier ion used (identification was based on full EI spectrum match)

Supplemental Table S3

Individual ETs performed and description of recipients, embryos, bull breed and pregnancy diagnosis to term in discovery studyand validation study with Holstein recipients.

			Recipient		Culture	Embryo stage		Bull		Thawing [(1)]	Pregnancy diagnoses		
Day-0	Study	ET	ID	Breed	to Day-6	Day-6	Day-7 blastocyst	ID	Breed	re-expansion	Day-40	Day-62	Birth
28/01/2014	Discovery	1	9	Holstein	BSA	Morula	Fully expanded	A	AV	3/3	Pregnant	Pregnant	To term
28/01/2014	Discovery	2	23	Holstein	BSA	Morula	Fully expanded	B	Holstein	3/3	Pregnant	Pregnant	To term
11/02/2014	Discovery	3	1	Holstein	BSA	Morula	Fully expanded	B	Holstein	2/2	Open	Open	Open
25/02/2014	Discovery	4	15	AV	BSA	Blastocyst	Fully expanded	D	Holstein	2/2	Pregnant	Pregnant	To term
18/03/2014	Discovery	5	6	AV	BSA	Morula	Expanding	A	AV	2/2	Pregnant	Pregnant	To term
03/06/2014	Discovery	6	4	Crossbred	BSA	Morula	Fully expanded	F	AV	4/4	Pregnant	Pregnant	To term
03/06/2014	Discovery	7	17	Holstein	BSA	Morula	Fully expanded	F	AV	4/4	Pregnant	Pregnant	To term
03/06/2014	Discovery	8	19	AV	BSA	Morula	Fully expanded	F	AV	4/4	Pregnant	Pregnant	Open
30/07/2014	Discovery	9	1	Holstein	BSA	Morula	Fully expanded	A	AV	2/2	Pregnant	Pregnant	To term
30/07/2014	Discovery	10	5	Holstein	BSA	Morula	Fully expanded	A	AV	2/2	Open	Open	Open

20/08/2014	Discovery	11	7	AV	BSA	Morula	Fully expanded	B	Holstein	2/2	Open	Open	Open
17/09/2014	Discovery	12	24	Holstein	BSA	Blastocyst	Fully expanded	A	AV	1/1*	Open	Open	Open
15/10/2014	Discovery	13	18	Holstein	BSA	Blastocyst	Fully expanded	F	AV	1/1	Open	Open	Open
12/11/2014	Discovery	14	3	Crossbred	BSA	Morula	Fully expanded	A	AV	2/2	Open	Open	Open
10/12/2014	Discovery	15	24	Holstein	BSA	Morula	Expanding	A	AC	1/1*	Pregnant	Pregnant	To term
21/01/2015	Discovery	16	19	AV	BSA	Morula	Fully expanded	A	AV	2/2	Open	Open	Open
10/06/2015	Discovery	17	2	AV	BSA	Blastocyst	Fully expanded	C	AV	1/1*	Open	Open	Open
11/09/2015	Discovery	18	26	Holstein	BSA	Morula	Fully expanded	C	AV	1/1	Pregnant	Pregnant	Open
15/09/2015	Discovery	19	25	Holstein	BSA	Blastocyst	Fully expanded	C	AV	2/2	Open	Open	Open
29/10/2015	Discovery	20	11	AV	BSA	Blastocyst	Fully expanded	D	Holstein	4/4	Pregnant	Pregnant	To term
29/10/2015	Discovery	21	16	Crossbred	BSA	Morula	Fully expanded	D	Holstein	4/4	Pregnant	Pregnant	To term
29/10/2015	Discovery	22	17	Holstein	BSA	Blastocyst	Fully expanded	D	Holstein	4/4	Pregnant	Pregnant	To term
29/10/2015	Discovery	23	27	AV	BSA	Blastocyst	Fully expanded	D	Holstein	4/4	Open	Open	Open
14/12/2015	Discovery	24	30	Holstein	BSA	Blastocyst	Fully expanded	C	AV	3/4	Open	Open	Open
14/12/2015	Discovery	25	31	Holstein	BSA	Blastocyst	Fully expanded	C	AV	3/4	Pregnant	Open	Open

14/12/2015	Discovery	26	32	Holstein	BSA	Blastocyst	Expanding	G	AV	3/4	Pregnant	Pregnant	To term
05/05/2016	Discovery	27	14	AV	BSA	Blastocyst	Fully expanded	C	AV	3/3	Open	Open	Open
05/05/2016	Discovery	28	28	AV	BSA	Blastocyst	Fully expanded	G	AV	3/3	Pregnant	Pregnant	To term
14/07/2016	Discovery	29	20	Crossbred	FCS+BSA	Morula	Expanding	C	AV	4/5	Pregnant	Pregnant	To term
14/07/2016	Discovery	30	22	Crossbred	FCS+BSA	Morula	Expanding	C	AV	4/5	Open	Open	Open
07/09/2016	Discovery	31	8	AV	BSA	Morula	Fully expanded	C	AV	4/5	Open	Open	Open
07/09/2016	Discovery	32	10	AV	BSA	Blastocyst	Fully expanded	C	AV	3/3	Pregnant	Pregnant	To term
07/09/2016	Discovery	33	21	Crossbred	BSA	Morula	Expanding	C	AV	3/3	Pregnant	Pregnant	To term
13/12/2016	Discovery	34	12	Holstein	BSA	Blastocyst	Fully expanded	E	Holstein	3/3	Pregnant	Pregnant	To term
13/12/2016	Discovery	35	13	AV	BSA	Blastocyst	Fully expanded	E	Holstein	3/3	Pregnant	Pregnant	To term
13/12/2016	Discovery	36	29	Holstein	BSA	Morula	Fully expanded	E	Holstein	3/3	Pregnant	Pregnant	To term
15/06/2017	Validation	37	35	Holstein	BSA	Blastocyst	Fully expanded	H	Holstein	3/3	Open	Open	Open
15/06/2017	Validation	38	40	Holstein	BSA	Morula	Expanding	H	Holstein	3/3	Pregnant	Pregnant	To term
15/06/2017	Validation	39	43	Holstein	BSA	Morula	Expanding	H	Holstein	3/3	Pregnant	Pregnant	To term
10/08/2017	Validation	40	41	Holstein	FCS+BSA	Blastocyst	Fully expanded	E	Holstein	3/3	Open	Open	Open

10/08/2017	Validation	41	42	Holstein	FCS+BSA	Blastocyst	Fully expanded	E	Holstein	3/3	Open	Open	Open
10/08/2017	Validation	42	43	Holstein	FCS+BSA	Blastocyst	Fully expanded	E	Holstein	3/3	Pregnant	Pregnant	To term
21/09/2017	Validation	43	38	Holstein	FCS+BSA	Blastocyst	Fully expanded	H	Holstein	2/3	Open	Open	Open
21/09/2017	Validation	44	39	Holstein	FCS+BSA	Blastocyst	Fully expanded	H	Holstein	2/3	Open	Open	Open
03/10/2017	Validation	45	42	Holstein	BSA	Blastocyst	Fully expanded	G	AV	1/2	Open	Open	Open
05/12/2017	Validation	46	38	Holstein	BSA	Morula	Expanding	I	Holstein	2/2	Pregnant	Pregnant	To term
05/12/2017	Validation	47	39	Holstein	BSA	Morula	Expanding	I	Holstein	2/2	Pregnant	Pregnant	Open
18/01/2018	Validation	48	37	Holstein	FCS+BSA	Blastocyst	Fully expanded	G	AV	2/2	Pregnant	Pregnant	To term
18/01/2018	Validation	49	46	Holstein	FCS+BSA	Blastocyst	Fully expanded	G	AV	2/2	Pregnant	Pregnant	To term
08/02/2018	Validation	50	33	Holstein	FCS+BSA	Blastocyst	Expanding	I	Holstein	1/1	Pregnant	Pregnant	To term
08/03/2018	Validation	51	34	Holstein	FCS+BSA	Blastocyst	Expanding	J	AV	3/3	Open	Open	Open
08/03/2018	Validation	52	36	Holstein	BSA	Morula	Fully expanded	J	AV	3/3	Pregnant	Pregnant	To term
08/03/2018	Validation	53	44	Holstein	FCS+BSA	Blastocyst	Fully expanded	J	AV	3/3	Pregnant	Pregnant	Open
04/04/2018	Validation	54	45	Holstein	BSA	Morula	Expanding	H	Holstein	3/3*	Pregnant	Pregnant	To term
04/04/2018	Validation	55	47	Holstein	FCS+BSA	Blastocyst	Fully expanded	H	Holstein	3/3*	Open	Open	Open

Recipients 1, 19, 24, 38, 39, 42 and 43 were transferred twice as heifers; recipient 17 was transferred once as heifer and a second time as uniparous, non-lactating cow.

AV: Asturiana de los Valles breed.

Crossbred recipients were used only in embryo culture medium studies, not plasma studies.

(1): N embryos that re-expanded in single culture prior to ET / N embryos thawed. Asterisks indicate unknown numbers of total embryos thawed.

Supplemental Table S4

Capacity of candidate metabolite biomarkers identified in embryo culture medium (CM) and Day-0 recipient plasma to predict pregnancy to term in Asturiana de los Valles recipients as single biomarkers and combined within a F1 score.

	CM	Day-0 plasma	Biomarker used		
Class	Capric acid	Heptadecanoic acid	Only CM	Only plasma	F1 Score
Birth	3447	250243	True	True	True
Birth	2547	267024	True	True	True
Birth	-1026	219331	True	True	True
Birth	-3649	237564	True	True	True
Birth	3586	270552	True	True	True
Birth	159	265988	True	True	True
**Lower limit**	**-3649**	**219331**			
**Higher limit**	**4392**	**270552**			
No birth	4579	297212	True	True	True
No birth	3282	347269	False	True	True
No birth	-718	259290	False	False	False
No birth	938	317382	False	True	True
No birth	4443	266121	True	False	True
No birth	4549	245545	True	False	True
No birth	1824	407111	False	True	True
Coverage	0.728	0.810			0.923

Range limits of pregnancy in culture medium calculated over 36 embryos transferred.

Metabolite levels are classified in recipients that reached birth and lower and higher limits within each metabolite are highlighted. Metabolites that do not correctly fit in the assigned classification are colored.

Coverage represents the ROC-AUC values for single metabolites, and F1 score showed as correctly classified samples.

Concentrations are relative values (arbitrary units).

Supplemental Table S5

Relative concentrations by targeted GC-MS (with separation of blastocyst and morula as Day-6 embryonic stages) of metabolites selected in embryo culture medium (CM) containing BSA or FCS+BSA subtracted from blanks (CM with no embryos), measured in N=19 samples corresponding to embryos transferred to Holstein recipients that differed in reaching pregnancy to term or not

CM	Day-6 stage	Pregnancy	N	Capric acid	Glycerol monoesterate
FCS+BSA	Early Blastocyst	Birth	4	-0.529±0.066	1.463±1.585
FCS+BSA	Early blastocyst	No birth	7	0.012±0.052	4.549±1.261
BSA	Early blastocyst	No birth	2	-0.187±0.086	0.746±2.083
BSA	Morula	Birth	5	0.069±0.058	2.846±1.410
BSA	Morula	No birth	1	-0.030±0.132	3.465±3.183
		P Value		0.270	0.577
Major effects				P values	
CM				0.123	0.164
Stage				0.106	0.173
CM*Stage				0.234	0.330
Pregnancy				0.911	0.244

Data are LSMean±SEM. No differences were observed

Supplemental Table S6

Embryo transfers with vitrified/warmed embryos and recipient breeds used in experiments and their pregnancy rates on Day-40, Day 62 and birth.

Purpose	Metabolomic Technique	Breed	N	Pregnancies (%)		
				Day-40	Day-62	Birth
Discovery [1]	GC-qTOF (untargeted)	Holstein	17	11 (65)	10 (59)	9 (53)
Discovery [1]	GC-qTOF (untargeted)	AV	13	7 (54)	7 (54)	6 (46)
Discovery [1,2]	GC-qTOF (untargeted)	Crossbred	6	4 (67)	4 (67)	4 (67)
Validation[3]	GC-qTOF (targeted)	Holstein	19	11 (58)	11 (58)	9 (47)

[1]: Discovery samples used to develop the models

[2]: Only embryo culture medium, not recipient plasma used from crossbred cattle.

[3]: Independent samples used to validate the modelswhich were obtained after metabolite selection within discovery samples

AV: Asturiana de los Valles.

N: ETs performed
